# A Novel Network Approach to Capture Cognition and Affect: COVID-19 Experiences in Canada and Germany

**DOI:** 10.3389/fpsyg.2021.663627

**Published:** 2021-06-11

**Authors:** Jordan Mansell, Lisa Reuter, Carter Rhea, Andrea Kiesel

**Affiliations:** ^1^Network for Economic and Social Trends, Western University, London, ON, Canada; ^2^Cluster of Excellence livMatS @FIT Freiburg Center for Interactive Materials and Bioinspired Technologies, University of Freiburg, Freiburg, Germany; ^3^Department of Psychology, Cognition, Action, and Sustainability Unit, University of Freiburg, Freiburg, Germany; ^4^Department of Physics, Université de Montréal, Montreal, QC, Canada

**Keywords:** COVID-19, cognition and affect, network approach, cognitive-affective mapping, Germany, Canada, network analysis

## Abstract

We tested a novel method for studying human experience (thoughts and affect). We utilized Cognitive-Affective Maps (CAMs)–an approach to visually represent thoughts and their affective connotations as networks of concepts that individuals associate with a given event. Using an innovative software tool, we recruited a comparative sample of (*n* = 93) Canadians and (*n* = 100) Germans to draw a CAM of their experience (events, thoughts, feelings) with the Covid-19 pandemic. We treated these CAM networks as a series of directed graphs and examined the extent to which their structural properties (latent and emotional) are predictive for the perceived coronavirus threat (PCT). Across multiple models, we found consistent and significant relationships between these network variables and the PCT in both the Canadian and German sample. Our results provide unique insights into individuals' thinking and perceptions of the viral outbreak. Our results also demonstrate that a network analysis of CAMs' properties is a promising method to study the relationship between human thought and affective connotation. We suggest that CAMs can bridge several gaps between qualitative and quantitative methods. Unlike when using quantitative tools (e.g., questionnaires), participants' answers are not restricted by response items as participants are free to incorporate any thoughts and feelings on the given topic. Furthermore, as compared to traditional qualitative measures, such as structured interviews, the CAM technique may better enable researchers to objectively assess and integrate the substance of a shared experience for large samples of participants.

## Introduction

Developed by the cognitive scientist and philosopher Paul Thagard, *Cognitive-Affective Maps* (CAMs) are a direct mental modeling approach for visually depicting the content of belief systems (Thagard, 2010). While CAMs contain several quantifiable properties, their feasibility as an empirical tool is yet to be tested. Using the COVID-19 pandemic as a test case, we explored the application of CAMs as a quantitative tool for the study of human experiences including thought and affective connotations. Specifically, we ask the question *can the network properties of CAMs be used to study similarities in individuals' thinking and experience surrounding an emotional life event?* To answer this question on the feasibility of CAMs, we recruited a sample of Canadian (*n* = 93) and German (*n* = 100) subjects to create CAMs encoding their experiences with the COVID-19 pandemic, we then assessed the resulting mental models, using a stepwise analysis of their network properties.

CAMs are visualized networks consisting of nodes and links that connect affective and cognitive elements. The nodes represent various content in text form, including goals, events, people, ideas or concepts, emotions, factual knowledge, or conclusions. Each node also conveys an emotional value, which is represented by the color and shape of the node. These valences or affects are broad assessments in terms of “positive” or “negative” and they can be related to emotion, mood, and motivation (Thagard, 2012b). CAMs also include links (sometimes called edges), that are lines connecting two nodes. Based on the computational theory of explanatory coherence (Thagard, 1989), there are two types of links between concepts in CAMs: supporting links and contradictory links. Applied to the study of attitudes and experiences, CAMs are a rich source of individual level data, however, the lack of an appropriate tool to encode and analyze this information is an ongoing constraint to their wider usage in research. Past applications of CAMs were limited to qualitative evaluations or discussions about the structure and function of belief systems (Thagard, 2010, 2011, 2012a,b,c, 2015, 2018; Wolfe, 2012; Homer-Dixon et al., 2013, 2014; Milkoreit, 2013; Findlay and Thagard, 2014; Luthardt et al., 2020). Within the published literature utilizing CAMs, exemplar CAMs were typically drawn by the researchers themselves, using a critical evaluation of a corpus of data (speech or written document, e.g., Luthardt et al., 2020). Unfortunately, when used exclusively, these qualitative methods are subject to several limitations including the introduction of subjective (reader) biases and the restriction to small sample sizes. In our opinion, the lack of a standardized method for the creation, analysis, and comparison of CAMs is significantly impeding their wider application across the social and psychological sciences. Fortunately, the increased accessibility of analytic tools for network analysis and the creation of online applications such as Valence^1^ means that it is possible to use CAMs to analyze and compare how individuals perceive a single issue or experience.

CAMs contain a number of significant opportunities not provided by other research methods. In contrast to questionnaires, CAMs can directly capture the connection between the elements of interest to a specific individual participant. Furthermore, because they use an open response visualization tool, as opposed to structured survey items, their content is less susceptible to instrument biases. For example, CAMs' less structured response format provides participants an opportunity to elaborate on factors which are potentially overlooked or discounted by structured response items. Compared to qualitative interviews, larger amounts of data can be collected with less effort as participants are able to draw the CAMs themselves and the data analyses does not require a detailed transcription of the interview. Finally, CAMs can be combined with survey, interview, and experimental methods to generate richer datasets, conduct robustness checks or cross method comparisons, and explore causal relationships.

We investigate the feasibility of using CAMs' network properties to study similarities in individuals' experiences with a shared emotional life event. To meet this objective, we administered an incentivized online study during the 2020 COVID-19 pandemic. We asked samples of Canadian and German participants to visually depict their experience with the ongoing viral out-break as a CAM, using the Valence application (Rhea et al., 2020). In detail, we asked participants to capture their experience, the events, thoughts, and feelings resulting from the current coronavirus outbreak and draw everything that comes to their mind concerning their experience with the coronavirus pandemic. We selected the pandemic as our case study to maximize the external validity in our assessment of individuals' experience (thought and affect). In contrast to laboratory manipulations which subjects often perceive of as artificial or unrelatable, the global nature of the pandemic emergence ensures that all individuals in our study have experienced some degrees of life disruption. Our design therefore relies on within sample variation on the perceived threat of coronavirus to test the predictive validity of CAMs as an empirical tool. Participants also completed demographic questionnaires capturing their experience with the viral outbreak, including a 3-item scale to assess perceived coronavirus threat (PCT) developed by Conway et al. (2020). For future research on the factors mediating or moderating the PCT, we administered questionnaires on the meaning of life (Breyer and Danner, 2015), control conviction (Kovaleva et al., 2012), need for cognition (Beißert et al., 2015), need for affect (Appel et al., 2012), and personal need for structure (Neuberg and Newsom, 1993). For future research on the social consequences of the PCT we also administered questions on socio-political attitudes.

To evaluate the predictive value of CAMs' network properties we operationalized and compared 14 network properties for each individual CAM (including centrality, density, average valence, and valence of central node) which capture both the emotional and latent properties of CAMs (Supplementary Table 6). Based on the principles of CAMs developed by Thagard (2010), our expectation is the emotional properties of CAMs should be correlated with the PCT. Specifically, participants who draw CAMs with a more negative valence should be more likely to perceive the coronavirus as threatening? Given the exploratory nature of the study, we have no expectations about the relationship between latent properties and PCT, however, a discussion of possible relationships is included in the Supplementary Material (page 9). Finally, we had no a priori expectations about the differences between Canadian and German samples.

In reporting our results, across multiple models, we found a consistent and significant relationship between these network variables and the PCT in both the Canadian and the German sample. However, in the German sample we found an unexpected relation between valence and PCT. Our results demonstrate that a network analysis of CAM properties is a promising method to study the relationship of the overall assessment of a situation e.g., in terms of threat and the detailed assessment of the experience in terms of thoughts/concepts and their affective assessment.

### Properties of CAMs

**Figure 1:**
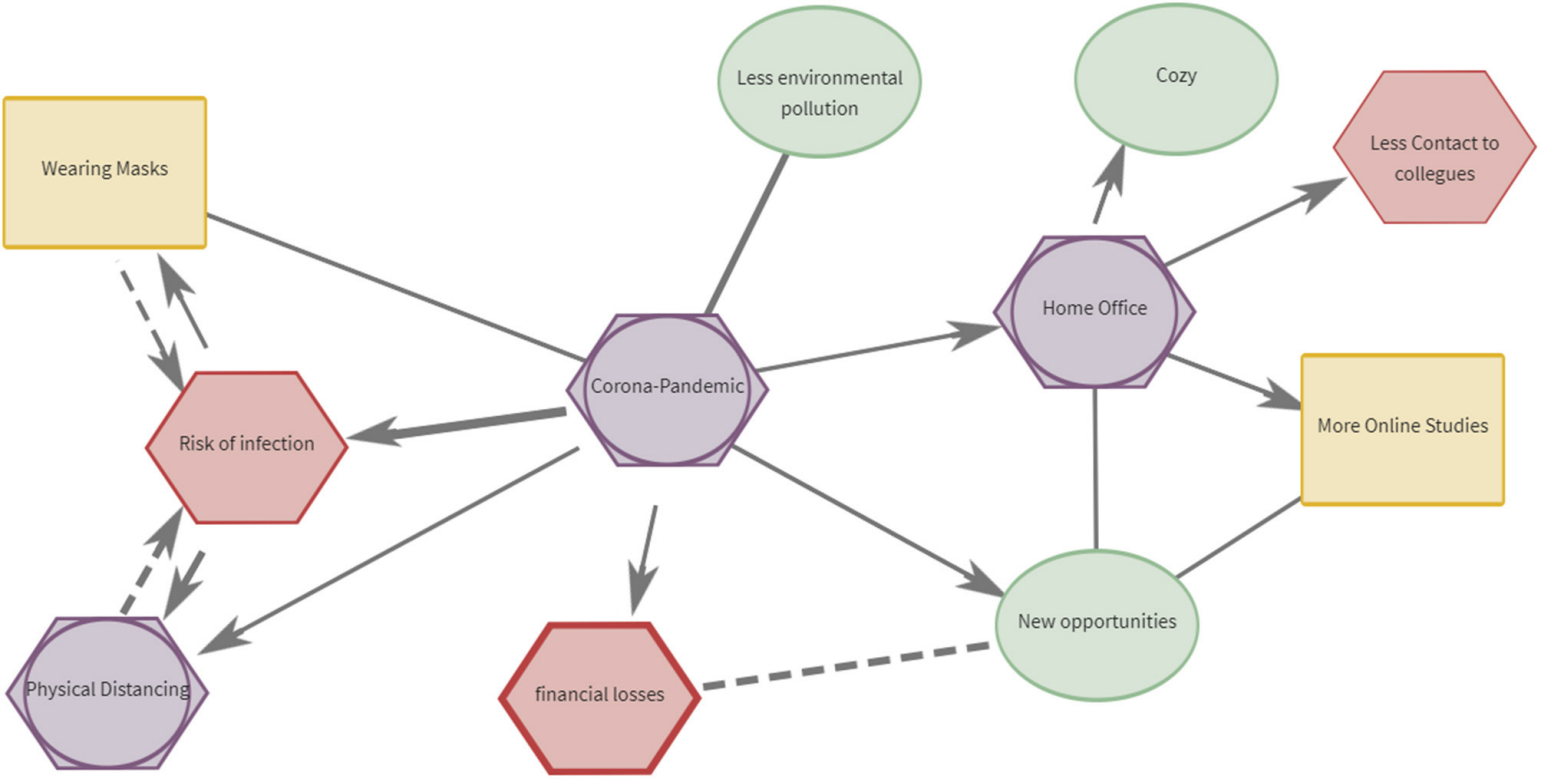
Exemplary CAM on the topic of the corona pandemic. Figure displays a summary of a CAM's properties: ambivalent (purple), negative (red), neutral (yellow), and positive (green) concepts. Solid lines indicate concepts which are mutually reinforcing, dashed lines indicate concepts which are oppositional. Thicker lines indicate stronger relationships. Arrows indicate the direction of a causal relationship between concepts. Two arrows between two concepts indicate a bidirectional or mutually reinforcing relationship. No arrows indicate there is no clear causal relationship.

Outlined in Thagard (2010), the rules for drawing a CAM are illustrated as follows (Figure 1 for an exemplary CAM). Every node in the CAM can be evaluated affectively. There are eight valence gradations to choose from, which are divided into four different colors and shapes. Green ovals stand for positive valence, red hexagons for negative valence. Nodes can be coded as neutral and ambivalent. Neutral, represented as a yellow rectangle, means that the node is not associated with positive or negative affect. Ambivalent, represented by a purple hexagon superimposed over a circle, means that the node contains a mixture of positive and negative values. There are three intensity levels for both positive and negative values–the thicker the border, the more intense the affect.

These different nodes are connected through lines (links or edges; in the following we use the term link to refer to these connecting lines). There are two different kinds of links to choose from: solid and dashed. Solid links indicate that the two elements are positively correlated, whereas dashed links mean that the two elements are negatively correlated. According to Thagard's (2010) rules for drawing CAMs, the links do not represent causal directional effects. In our study, we modified the original format to allow participants to include arrows as a link property. We interpret these arrows as an indicator of a directed relationship where one concept contributes to the occurrence of another following the direction of the arrow e.g., A contributes to B if A → B. The outcome of this modification is that for the purpose of statistical analysis CAMs can be treated as directed (Markov) graph which allows us to generate a wider variety of network measures. These measures are valuable as they allow us to explore how different structural relations within the data contribute to a given perspective.

### Exploratory Questions and Expectations

The COVID-19 pandemic has brought about a global health emergency (Sohrabi et al., 2020) broadly affecting people around the world. Responses to the pandemic such as social distance, self-isolation, travel restrictions, and school closures have far-reaching effects on work, family life and leisure time behavior (Nicola et al., 2020). Reviews of COVID-19 studies show a considerable increase in anxiety, stress and depression levels and sleep disturbance (Rajkumar, 2020; Salari et al., 2020; Sandín et al., 2020; Usher et al., 2020; Vindegaard and Benros, 2020). Because of these severe and widespread consequences, the pandemic represents an ideal case to study human experience (thought and affect).

While the consequences of the pandemic vary by region and by person, the global characteristic of the pandemic means that all individuals, to varying degrees, are administered treatment. Our study exploits the variation in outcomes to study the relationship between individuals' structured thoughts and experience. In other words, we investigate whether similarities in individual structured thinking are predictive of similarities in individual experience.

In this study, we ask the following question: *Can the network properties of Cognitive Affective Maps be used to study similarities in individuals' thinking and experience surrounding an emotional life event, the 2020 COVID-19 pandemic?* To answer this question, we evaluated the following exploratory questions about the relationship between CAMs' network properties and the perceived threat of COVID-19 assessed by the 3-item PCT scale of Conway et al. (2020). A justification of these questions is provided in Supplementary Material (“Exploratory Question Rational”).

### Exploratory Questions

1. Do the emotional network properties of CAMs (e.g., average valence and valence of central node) predict the perceived threat of the coronavirus?
2. Do the latent network properties of CAMs (e.g., density, diameter, closure) predict the perceived threat of the coronavirus?
3. If so, to what extent are the network properties that predict the perceived threat of the coronavirus consistent across samples?

## Method

### Sample

Initially, we recruited (*n* = 106) Canadian and (*n* = 110) German participants from Prolific, an online participants recruitment tool for academic research. Due to the exploratory nature of the study, two independent samples (Canadian and German) are used to increase the generalizability and reliability of our findings. Study sample size was set by the availability of research funds. To demonstrate that our study is sufficiently powered, a separate one-sided *post-hoc* power analysis using the results of the conditioned statistical model (Model 2) is conducted for Canadian and German samples. Summarized in Supplementary Tables 80, 81, the results show that there is sufficient power to detect each of the significant effects but it is underpowered in the case of marginal effects.

During data cleaning, the data of 12 participants were dropped because they indicated that they prematurely stopped the CAM exercise, 11 additional participants were dropped for failing an attention check. For the purposes of the network analysis, we also restricted observations to participants whose CAM contain a minimum of three nodes or two links, this results in the exclusion of 1 additional participant. The final sample is (*n* = 93) Canadians and (*n* = 100) Germans. Due to the 2020 coronavirus pandemic, it was necessary to conduct our data collection online. The Prolific data collection platform was selected because it provides transparent information about the demographic composition of its subject pools by country and because it maintains subject pools in both Canada and Germany, representing the nationalities of the research team. The sample was restricted to Canadian and German residents over the age of 18 who had access to a laptop, notebook, or desktop computer. We further restricted participation to participants who speak fluent English (in Canada residents) or fluent German (in German residents). To incentivize participation, each participant was given a direct £8.50 payment for their participation. British pounds are the standard currency on Prolific, this payment converts to $14.50 CAN/ €9.50 GER. Participant compensation is based on an hourly minimum wage in Canada and Germany. The estimated length of the study was indicated with 60 min. Most participants completed the study in <60 min.

Data collection for this study was administered in two waves, the first from May 28th to May 29th (1), and June 21st to June 24th (2), 2020. During this time number of cases per 100,000 inhabitants differed greatly. According to Johns Hopkins University Medicine (2020), during the data collection periods the number of reported cumulative infection cases were as follows: Canada—May 28th [99,976], June 24th [104,087]; Germany—May 28th [182,196], June 24th [192,871]. In our sample, there are no significant correlations between time of data collection and the PCT (Supplementary Tables 7, 8).

The completion rates, measuring all participants who opened the study as compared to participants who completed the study, were 61.8% (CAN) and 64.4% (GER). All demographic data and questionnaire measures (Supplementary Tables 9–27) were collected after the CAM exercise. After the collection of demographic data, we fully randomized the order of attitudinal and psychological measures and the presentation of all questions within these measures.

### Demographic Summary

The Canadian sample (*n* = 93) is 44.09% female, 53.76% male, 1.00% non-binary, and 1.00% prefer not to say. The mean age category of the sample is 26–32 years (SD = 1.17), and it is 58.84% White, 45.16% other groups; 58.06% of the sample obtained a minimum of a college undergraduate, and 48.08% of the sample identifies religion as being “Very important” or “Somewhat important” in their life. The German sample (*n* = 100) is 34.00% female, 65.00 % male, 1.00% non-binary. The mean age is 26–32 (SD = 0.83), 76.00% of the sample indicate that both of their parents are of German ancestry, 12.00% have at least one parent of German ancestry, and 12.00% have no parent of German ancestry; 80.00% of the sample obtained a minimum of a college undergraduate, and 24.00% of the sample identifies religion as being “Very important” or “Somewhat important” in their life.

### Attitudinal and Psychological Measures

We administered a series of psychological measurements of the impact of COVID-19 developed by Conway et al. (2020). Drawing a CAM is a time intensive exercise (~30 min), to minimize survey fatigue we choose to administer short survey batteries whenever possible. The short version battery of Conway et al. (2020) consists of the following questionnaires: (1) *Perceived Coronavirus Threat* (PCT) Questionnaire with three items^2^, (2) Governmental Response to Coronavirus Questionnaire with six subscales with two items each, (3) Coronavirus Impacts Questionnaire with three subscales with two items each, and (4) Coronavirus Experience Questionnaire with three subscales and seven items each (Supplementary Tables 18–26). We focus on the three item 7-point measure of PCT, Canada (α = 0.775), Germany (α = 0.884). We also administered a 10-item measure of *need for affect* (Appel et al., 2012) which captures individuals' differences in the need to approach or avoid emotions (Canada [α = 0.794], Germany [α = 0.830]), and a 6-item measure of *need for structure* (Neuberg and Newsom, 1993), which measures personal need for a structured, simple, and predictable environment Canada (α = 0.840), Germany (α = 0.806) (Supplementary Table 27). For readers not familiar with these scales, please note that for each measure larger measured values indicate more perceived threat, need for affect or need for structure.

### CAMs–Network Properties

In total we operationalized 14 network properties, using the *CAM Network Analysis* tool (Rhea et al., 2021). These properties can be divided into two categories, emotional and latent. Emotional properties measure how the valence of individual nodes contributes to the overall CAM (Average Valence, Percentage of each node type, Central Node Valence). Latent properties refer to the number of nodes, links, and their interconnectedness (Centrality, Density, Diameter, Number of Nodes, Number of Links, triadic Closure). A definition of all network measures is listed in Supplementary Table 6. Of note, average valence incorporates the strength of an emotion weak—strong on a three-level categorical scale. This differs from the percentage of valence nodes (ambivalent, negative, neutral, positive) which only consider the number of nodes with a given valence relative to all other nodes. The properties and Pearson's correlations of these measures are summarized in Supplementary Tables 30–38.

### Procedure

Participants drew their CAM using the Valence online application, an editable graphic space: https://cam1.psychologie.uni-freiburg.de/users/loginpage?next=/. Participants began the CAM-drawing exercise by reviewing a set of neutral visual instructions which guided them through the process of drawing a CAM using the online application (Supplementary Material “Instructions”). Participants were able to keep the CAM-drawing instructions open during the exercise. After completing the CAM instructions, we asked participants to draw a CAM which captures their experiences with the coronavirus outbreak. We used the wording:

*We are interested in capturing your experience, the events, thoughts, and feelings, resulting from the current coronavirus outbreak. Using the mapping tool, please draw everything that comes to mind concerning your experience with the coronavirus. Think about what matters in the current coronavirus outbreak and please do your best to draw everything that comes to your mind concerning the coronavirus*.

Participants were also instructed not to spend more than 30 min on the mapping exercise. The full instructions as presented to all participants are listed in the Supplementary Material (“Instructions”). The instructions are also available via OSF: https://osf.io/8mxcz/?view_only=750d8048ed6a4c629d03f11bcc03c454.

After completing their CAMs, participants answered a set of control questions and four attention checks which measured whether they correctly understood the properties associated with CAMs. The data of participants who answered incorrectly to more than one of these four questions were dropped from the analysis. The instructions for the study were originally written in German and then translated into English. The instructions were reviewed and edited by two native German and two native English speakers prior to data collection. After drawing the CAMs, participants completed all demographic and further questionnaire measures.

### Approach to Analysis

To investigate whether the structural properties of individuals' CAMs are correlated with PCT we used the general linear statistical model (GLM) with robust standard errors. A GLM is used, as opposed to an Ordinary Least-Squares (OLS), to account for the non-normal distribution of errors in several network measures as well as the non-normal distribution of the measures themselves. The dependent variable in these regressions is the standardized measure of the PCT. For each regression we reported the Akaike Information Criterion (AIC) and Residual Deviance (D), deviance adjust for degrees of freedom. The independent variables are the emotional and latent network measures (Supplementary Table 6). To minimize skewedness and kurtosis in specific variables, we performed a logistic transformation on 6 measures: (i) density, (ii) number of nodes, (iii) number of links, (iv) number of contradicting links, (v) number of supporting links, (vi) triadic closure. To better compare their relative effect sizes all network variables are standardized.

As an exploratory study, we had limited evidence on which to base assumptions about the expected significance of each network measure. Consequently, we conducted our regression model using a stepwise approach. We began by including all network measures as covariates and then iteratively dropping the item with the lowest significance until all terms in the model are meaningful. Following Hosmer Jr et al. (2013) a meaningful covariate is one whose *p*-value is at, or below, *p* ≤ 0.250. In addition to social and demographic characteristics Canada and Germany also differ in several direct factors which may influence the perceived threat of COVID-19 include the number of active cases, spread of the pandemic, and government response. To take account of these differences, we report our results with combined and separate Canadian and German samples. Cluster robust standard errors by country are used in the combined sample.

As a robustness measure, we ran the stepwise analysis with conditioned and unconditioned models for both the combined (German and Canadian) and the separate samples, these were labeled models 0, 1, 2, and 3. Model 0 was applied only in the combined sample and displayed the unconditioned correlations between the network measures and the PCT. Model 1 displayed unconditioned correlations between the network measures and the PCT, expect in the combined sample where model 1 included a control covariate for country. Model 2 included control covariables for age, education, gender. Model 3 included controls for: (i) need for affect, and (ii) need for structure. Need for affect and need for structure were included to rule out the possible mediating effects of traits previously associated with the sensitivity to threat or to life disruptions. In models 0, 1, 2, and 3, the control covariates were retained regardless of whether they met the *p* ≤ 0.250 significant threshold.

## Results

We found several consistent and significant correlations between network measures and the PCT. We focus on summarizing the meaningful and significant results with the full pattern of results reported in the Supplementary Tables 39–52. In each section, we report the result of the emotional network measures followed by the latent network measures. Of note, there is a significant difference in the PCT between samples (Supplementary Table 53), with German participants reporting lower levels of PCT than Canadians

(Coefficient = −0.606; Std. 0.137; *p* < 0.0001).

### German and Canadian Sample Combined

As summarized in Table 1, consistent with expectations, across models 0–3 *average valence* was negatively and significantly correlated with the PCT indicating that the more positive a CAM the lower the PCT. In models 0, 1, and 2 the *percentage of negative nodes* was retained and negatively correlated with PCT. This second result is contrary to expectations that more negatively valenced CAMs will positively correlate with PCT, however this variable only reaches significance in model 1, and drops out completely in model 3.

**Table 1:** General Linear Regression Model (GLM) with Robust confidence intervals in combined sample.

**Variable**	**Model 0**	**Model 1**	**Model 2**	**Model 3**
Average valence	−0.217^**^ (0.088)	−0.281^**^ (0.091)	−0.231^**^ (0.084)	−0.151^***^ (0.012)
Central node valence				
Centrality	0.166^***^ (0.10)	0.078^***^ (0.006)		0.093^***^ (0.012)
Density	−0.174^**^ (0.058)	−0.159^**^ (0.070)		−0.217^***^ (0.059)
Diameter			−0.104^†^ (0.058)	
Number nodes			0.204^***^ (0.009)	
Number links				
Number dashed				
Number solid				
Percentage ambivalent				
Percentage negative	−0.120 (0.078)	−0.165^**^ (0.078)	−0.114 (0.072)	
Percentage neutral				
Percentage positive				
Triadic closure				0.109^**^ (0.055)
Age			−0.054^**^ (0.024)	−0.048^**^ (0.021)
Education			−0.017 (0.026)	−0.002^**^ (0.021)
Gender				
Female			−0.360^**^ (0.135)	−0.283 (0.181)
Non-binary			−1.084^***^ (0.272)	−1.391^**^ (0.508)
Pref not say			1.503^***^ (0.255)	1.478^***^ (0.226)
Country		−0.654^***^ (0.028)	−0.652^***^ (0.030)	−0.634^***^ (0.048)
Need for affect				0.030 (0.099)
Need for structure				0.209^***^ (0.025)
Constant	0.000 (0.303)	0.339 (0.014)	0.800 (0.146)	0.649 (0.351)
N	193	193	193	193
Residual *df*	192	192	192	192
Scale parameter	0.987	0.890	0.855	0.821
Residual D	0.967	0.867	0.810	0.770
AIC	2.809	2.700	2.633	2.581

*Correlation between network measures and a standardized measure of the perceived threat of coronavirus in combined Canadian and German samples*.

† *p <0.100*.

** *p <0.050*.

*** *p <0.001*.

Looking at the latent measures, in models 0, 1, and 3, *centrality* was positively and significantly correlated with the PCT. This indicates that participants whose CAMs' structure is more dependent upon their central node (centralized), are more likely to perceive the virus as threatening. Similarly, in models 0, 1, and 3 *density* was negatively and significantly correlated with the PCT, indicating that higher levels of interconnectedness are associated with lower PCT. In model 2, the *number of nodes* was positively and significantly correlated with the PCT however, this variable was not retained in any of the other models. Finally, in model 3, *triadic closure* was positively and significantly correlated with PCT.

Overall, these results support the conclusion that latent properties are meaningful predictors of individual experience, however, the exact nature of this relationship remains unclear. For example, the observation that *density*, an alternative measure of connectivity, had an opposing relationship to PCT as compared to both *number of nodes* and *triadic closure* is important to the interpretation of our results. We discuss the limitation of the density measure in our discussion section.

### Canadian Sample

As expected, in models 1 and 2 *average valance* was negatively and significantly correlated with PCT (see Table 2), indicating that participants who drew more positive CAMs were less likely to perceive the coronavirus as threatening. In model 3, *average valence* was dropped from the model and replaced by the *percentage of positive nodes* which was also negatively and significantly correlated with the PCT. Here, the dropping of *average valence* was a consequence of the stepwise process and the initial inclusion of multiple correlated covariates into a single overfit model. As shown in Supplementary Tables 51, 52, when *percentage of positive nodes* was replaced in the final model by *average valence*, average valence was significant at the 95% confidence level. It is also worth noting that the two variables, *average valence* and *percentage of positive nodes* where highly correlated (Pearson's correlation: 0.823, *p* < 0.0001), (Supplementary Tables 33–35). Lastly, contrary to expectations, the *percentage of negative nodes* in models 1–3 was negatively correlated with the PCT, indicating that the greater the number of negative nodes the lower the level of PCT. As with the same effect in the combined sample this effect reached significance in models 1 and 2.

**Table 2:** General Linear Regression Model (GLM) with Robust confidence intervals in separated samples.

**Variable**	**Model 1 Canada**	**Model 1 Germany**	**Model 2 Canada**	**Model 2 Germany**	**Model 3 Canada**	**Model 3 Germany**
Average valence	−0.418^**^ (0.132)		−0.399^**^ (0.139)			
Central node valence						
Centrality	−0.151 (0.122)					
Density		0.343^†^ (0.194)		0.332^†^ (0.193)		0.342^†^ (0.192)
Diameter	−0.326^**^ (0.127)		−0.223^†^ (0.117)		0.188 (0.119)	
Number nodes	0.649^**^ (0.225)	0.450^**^ (0.166)	−0.913^***^ (0.303)	0.444^**^ (0.177)	−0.760^***^ (0.316)	0.429^**^ (0.168)
Number links	−0.490^**^ (0.225)		1.092^**^ (0.275)		0.931^**^ (0.080)	
Number dashed						
Number solid						
Percentage Ambivalence						
Percentage negative	−0.292^**^ (0.123)		−0.295^**^ (0.128)		−0.097 (0.080)	
Percentage neutral						
Percentage positive					−0.259^**^ (0.091)	
Triadic closure			0.211^†^ (0.116)		0.185 (0.115)	
Age			−0.111^†^ (0.067)	−0.099 (0.126)	−0.101 (0.063)	−0.064 (0.132)
Education			−0.046 (0.060)	0.001 (0.160)	−0.046 (0.059)	0.087 (0.144)
Gender						
Female			−0.415^**^ (0.171)	−0.239 (0.219)	−0.340^**^ (0.168)	−0.099 (0.220)
Non-binary			−1.843^***^ (0.338)	−0.763^†^ (0.398)	−2.083^***^ (0.415)	−0.879^**^ (0.385)
Pref not say			1.821^***^ (0.343)		1.752^***^ (0.359)	
Need for affect					0.163^†^ (0.095)	−0.070 (0.094)
Need for structure					0.160^†^ (0.091)	0.242^**^ (0.093)
Constant	0.371 (0.090)	−0.333 (0.098)	1.165 (0.453)	0.079 (1.063)	1.102 (0.448)	−0.593 (0.971)
N	93	100	93	100	93	100
Residual *df*	86	97	83	94	81	92
Scale parameter	0.754	0.953	0.671	0.968	0.654	0.920
Residual D	0.754	0.953	0.655	0.957	0.638	0.910
AIC	2.628	2.819	2.516	2.853	2.508	2.820

*Correlation between network measures and a standardized measure of the perceived threat of coronavirus in combined Canadian and German samples*.

† *p <0.100*.

** *p <0.050*.

*** *p <0.001*.

Looking at the network properties, the *number of nodes* was positively correlated with the PCT in models 1–3. This indicates that participants who included more content into their CAMs were more likely to perceive the virus as threatening. In models 1–3 the *number of links* is negatively correlated with the PCT. This indicates that from a certain perspective the interconnectedness of a network is negatively correlated with the PCT, however, a limitation of this measure is that it does not correct for the total number of possible links. By comparison, *triadic closure*, which does correct for the total number of possible links, was positively correlated with the PCT, however, this measure was only retained in models 2 and 3, and was only marginally significant in model 2. Finally, in models 1–3 *diameter* is negatively correlated with the PCT, indicating that participants, whose networks are more expansive, are less likely to see the coronavirus as threatening. However, this term fails to reach significance in model 3. In summary, the results indicate that the latent properties of CAMs are a meaningful predictor of individual experience. Specifically, for the Canadian sample we see that CAMs that are expansive and more interconnected are associated with a greater perceived coronavirus threat. However, the relationship is not straight forward as the opposite relationship is observed in CAMs of the German sample.

### German Sample

In contrast to the combined and Canadian sample, no significant correlations were observed between the emotion-oriented network measures and PCT in the German sample. Looking at the latent measures, the *density* of the network and the *number of nodes* were both positively correlated with the PCT in model 1–3. While density remained marginally significant across all three models, the number of nodes reached significance at the 95% confidence level. Consistent with the combined and Canadian samples, this indicates that the amount of content and the interconnectedness of this content are significantly associated with the PCT in Germany. Taken together, the results provide good evidence that network properties of CAMs capture information significant to the larger study of individual experience (thought and connotation).

### Additional Regressions: Interaction Between Emotional and Latent Network Measures

Noted in the discussion of our research questions (Supplementary Material, page 9), one data trend which may be observed in participants' CAMs is the interaction between emotional and latent network properties. Drawing similarities from research in human memory, it is possible that the PCT will be associated with the interaction between density (high density) and valence (negativity). To answer this question, we ran a regression with first-order interactions of the emotional and all the latent network measures (Tables 2–5). Again, we applied a stepwise approach, however, because of the large number of interactions, we used a stricter criterion of *p* < 0.100. The same *p* ≤ 0.250 retention criterion was applied to individual covariates, except where a covariate contributed to a significant interaction. In general, we found similarities between the initial and interaction models, with both models including the same basic covariates. The largest difference was the retention of variables not included in the three previous regression models.

**Table 3:** General Linear Statistical Model (GLM): interaction between emotional and latent network and the standardized measure of the perceived threat of coronavirus.

**Variable**	**Coefficient**	***SD***	**Z**	**P>z**	**95% CI**
Average Valence	−0.228	0.161	−1.410	0.158	[−0.544, 0.089]
Centrality	0.674	0.080	8.380	0.0001^***^	[0.516, 0.831]
Central node value	0.043	0.017	2.560	0.010^**^	[0.010, 0.076]
Density log	−0.418	0.151	−2.760	0.006**	[−0.715, −0.122]
Number nodes log	−0.354	0.012	−28.690	0.0001^***^	[−0.378, −0.329]
Number links log	0.321	0.189	1.700	0.090^†^	[−0.049, 0.691]
Percentage negative	−0.131	0.080	−1.630	0.103	[−0.289, 0.026]
Percentage ambivalent	−0.015	0.001	−14.280	0.0001^***^	[−0.017, −0.013]
Centrality # central node value	0.234	0.024	9.550	0.0001^***^	[0.186, 0.282]
Density # central node value	0.086	0.044	1.950	0.051†	[0.000, 0.172]
Country	−0.674	0.005	−132.820	0.000	[−0.684, −0.664]
Constant	−0.016	0.051	−0.310	0.753	[−0.115, 0.083]
N					193
Residual *df*					192
Scale P					0.842
Residual D					0.794
AIC					2.613

*Combined sample*.

† *p <0.100*.

** *p <0.050*.

*** *p <0.001*.

**Table 4:** General Linear Statistical Model: interaction between emotional and latent network and the standardized measure of the perceived threat of coronavirus.

**Variable**	**Coefficient**	***SD***	**Z**	**P>z**	**95% CI**
Average valence	−0.371	0.118	−3.140	0.002^**^	[−0.603, −0.139]
Diameter	−0.198	0.098	−2.030	0.042^**^	[−0.390, −0.007]
Number links	−0.865	0.282	−3.070	0.002^**^	[−1.417, −0.312]
Number solid	0.364	0.178	2.050	0.040^**^	[0.016, 0.712]
Number nodes	0.823	0.201	4.090	0.0001^***^	[0.428, 1.217]
Percentage negative	−0.219	0.110	−1.980	0.048^**^	[−0.435, −0.002]
Central node value	0.102	0.100	1.020	0.307	[−0.094, 0.299]
Number nodes # percentage negative	−0.269	0.092	−2.920	0.004^**^	[−0.451, −0.088]
Number nodes # central node value	−0.337	0.088	−3.820	0.0001^***^	[−0.510, −0.164]
Constant	0.351	0.087	4.030	0.0001	[0.180, 0.521]
N					93
Residual *df*					83
Scale P					0.662
Residual D					0.662
AIC					2.526

*Canadian sample*.

† *p <0.100*.

** *p <0.050*.

*** *p <0.001*.

**Table 5:** General Linear Statistical Model: interaction between emotional and latent network and the standardized measure of the perceived threat of coronavirus.

**Variable**	**Coefficient**	***SD***	**Z**	**P>z**	**95% CI**
Density	0.388	0.250	1.550	0.121	[−0.103, 0.878]
Number nodes	0.566	0.221	2.560	0.010**	[0.133, 0.998]
Triadic closure	−0.044	0.120	−0.360	0.717	[−0.279, 0.192]
Central node value	0.100	0.099	1.000	0.315	[−0.095, 0.294]
Density log # central node value	0.793	0.255	3.110	0.002**	[0.293, 1.293]
Number nodes log # central node value	0.495	0.229	2.160	0.031**	[0.046, 0.944]
Triadic closure # central node value	−0.530	0.120	−4.430	0.0001***	[−0.765, −0.296]
Constant	−0.341	0.089	−3.820	0.0001	[−0.516, −0.166]
N					100
Residual *df*					92
Scale P					0.881
Residual D					0.881
AIC					2.788

*German sample*.

†p <0.100. **p <0.050. ***p <0.001.

#### Combined Sample

Looking at the combined sample, two interaction terms were retained. First, there was a positive and significant interaction between *centrality* and *valence of the central node*. Contrary to expectations, this indicates that as networks become more dependent on the central node and more positive, the PCT increases. Second, the interaction between *density* and *valence of the central node* was positive and marginally significant. Once again contrary to our expectations, this indicates that as CAMs become denser (more interconnected) and more positive, the greater the PCT.

#### Canadian Sample

Looking at the Canadian sample, there was a significant negative correlation between *number of nodes* and *percentage of negative nodes*. Difficult to interpret, this indicates that as the number of nodes and percentage of negative nodes increase, PCT decreases. Also observed was a significant negative correlation between *number of nodes* and *valence of the central node*. Consistent with our expectations this indicates that as the number of nodes increases, and the central node becomes more positive, the lower the PCT.

#### German Sample

Three interactions are observed in the German sample. First, there is a significant positive interaction between *density* and *valence of the central node*. Once again, and contrary to expectations, this indicates that as CAMs become more interconnected and the central node becomes more positive the PCT increases. Second, and contrary to expectations, there is a positive interaction between *number of nodes* and *valence of the central node* indicating that as the number of nodes increases, and the valence of the central node becomes more positive the greater the PCT. This is the opposite correlation as observed in the Canadian sample. Third, consistent with our expectations there was a negative interaction effect between *triadic closure* and *valence of the central node* indicating that as CAMs become more connected and the central node becomes more positive the lower the PCT.

## Discussion

Using the COVID-19 pandemic as a case study, we tested CAMs as a novel method for studying human experience (thought and affect). Specifically, we asked whether *the network properties of CAMs (emotional and latent) can be used to study similarities in individuals' thinking and experience surrounding an emotional life event?* To meet our objective, we operationalized and compared 14 network properties for each individual CAM. Our results yielded several interesting and significant network properties.

One immediate observation was a difference in significant emotional network measures between the Canadian and German sample. Looking at Table 2, in the Canadian sample, we found significant emotional and latent measures, while in the German sample we found only significant latent measures, yet no significant emotional measures. We conjecture that the German and Canadian sample differ regarding the emotional assessment of the COVID-19 pandemic. This assumption is backed up by our observation that scores on the PCT were significantly lower in the German than the Canadian sample. Please note that this finding is in line with other studies examining fearful reactions to the pandemic that also observed differences between countries (e.g., Lippold et al., 2020; Gruchoła and Sławek-Czochra, 2021). Lippold et al. (2020) for example reported that in terms of perceived fear of coronavirus Germany scores lowest among 96 countries, along with Austria and Sweden. Currently, we can only speculate why this is the case. It might be that variations in fear responses are a result of differences in government restrictions. However, data collected by University of Oxford^3^, tracking restriction measures during the pandemic worldwide shows that the restrictions in Canada and Germany did not meaningfully differ in the first half of 2020. According to these data, measures in Germany tended to be tightened earlier than in Canada, but the two countries quickly converged and as of May 2020 (prior to our data collection) both countries are listing in the same category for stringency of government response (index 50–75). Still, even if the restrictions were outwardly similar in severity, they could be perceived differently in each country. A first pointer to this is provided by a look at the central nodes of the CAMs and their affective assessment: Supplementary Tables 82, 83 show a categorization of the central CAM concepts, according to which in Canada concepts such as “Quarantine” are put in the center more frequently, while in Germany terms like “Restrictions” are more frequently referred to as central node (of course like expected the most frequent category in both countries is “Pandemic” or “Coronavirus”). Yet, to make more concrete statements about different perceptions, the content of all CAM concepts would have to be examined more closely, which is beyond the focus of this study. Another explanation for the differences in PCT between the countries refers to cultural differences related to workplace protection. In comparison to Canada, Germany has stricter employment projection protections (Chen and Hou, 2019). The German state has implemented a worker protection program which protects workers from losing their job during the pandemic and enables them to work in short time instead. By comparison, the Canadian Federal government extended its unemployment program to provide financial assistance to workers laid off because of the pandemic but did not guarantee job security. This difference in public policy may contribute to the differences in PCT observed in this study. This assumption is supported by research by Gruchoła and Sławek-Czochra (2021), who did not find a high fear of job loss, unemployment, reduction or loss of income for Germans, but did find these effects in other European countries. Unfortunately, a limitation of the present study is that it lacks the necessary measures to understand these baseline differences regarding perceived threat of COVID-19. Future research on CAMs using multiple samples will need to give greater attention to how contextual factors affect the salience or significance of an emotional event.

Furthermore, as described in the Supplementary Material, German CAMs also show less variation on the percentage of positive and negative nodes and the average valence of their CAMs. This suggests that as compared to Canadian participants, the coronavirus pandemic is a less negative emotional experience for Germans. To test this, we explored the interactions between country and different percentages of nodes and average valence as a predictor of PCT. Listed in Supplementary Tables 50–54, the results show that as the percentage of positive nodes and average valence of the CAM increased, Germans showed a greater PCT than Canadians. Additionally, as the percentage of negative, neutral and ambivalent nodes and the valence of the central node increased, Germans were less likely to perceive PCT than Canadians (Supplementary Tables 54–60). A psychological interpretation of these network correlations is difficult without analyzing the content of the CAMs. For example, the positive nodes of a CAM could be negatively correlated with the pandemic and explain a higher PCT. Such analyses are beyond the scope of this study. Future studies should therefore consider a mixed method approach which incorporate content-based automated analyses. Despite these differences in the Canadian and German sample, our results may at least provide tentative answers to two of the three research questions. Taking these differences into account, we argue the answer to questions 1 is yes: The emotional properties of CAMs did capture the perceived threat of the coronavirus. In both the combined and Canadian sample, average valence reliably predicted PCT. While other measures, such as the percentage of different nodes (e.g., positive, or negative) were not consistent, we believe these deficiencies were likely due to structural limitations within these variables and the differences between samples. The ability of participants to select four nodes with six difference valences introduced a large amount of variation in the percentages of nodal valence. The result was that the percentages of nodal variances tended not to be normally distributed, displaying skewedness or kurtosis (Supplementary Table 60). By contrast, the measure of average valence of the CAM was calculated using both the valence and strength scores for nodes and resulted in a network property with normal distribution. Lastly, the valence of the central node did not appear to be a significant variable except when interacted with the measures of network centrality, density, number of nodes, and triadic closure. This result indicates that as the networks become larger or more interconnected, the valence of the central node becomes a more important predictor of PCT. We assume this effect could also be influenced by the instructional setting–in the instructional example, the central node (shopping at the market) remains neutral. In total, 51% (*n* = 99) of the sample coded their central node as neutral, including 52 participants whose central node referenced the coronavirus, and a further 14 participants whose central node referenced restrictions, social distance, or stress concepts which might be otherwise expected to be defined as negative (see Supplementary Table 83 for the frequencies of affective assessments separately for both samples). To avoid this possible framing effect, future research utilizing CAMs will need to be explicit in informing participants that they are free to adjust the valence of all nodes in their CAM.

Turning to the latent network properties, we argue that the answer to question 2 is yes. Latent network properties captured the perceived threat of the coronavirus. All three samples show positive correlations between the *number of nodes* and the PCT. Furthermore, except for density in the combined sample, measures of network interconnectivity were also positively correlated with PCT. In other words, the results suggest an association between the salience or effect of an emotional event and levels of cognition and cognitive complexity. We note that while several other measures (centrality, diameter, triadic closure) were only periodically significant, these measures were highly correlated (Supplementary Tables 30–32) with the measures of density, number of nodes, and number of links, so it is not surprising that all the latent measures are not retained within the final model.

This is consistent with research on memory which demonstrates an association between the significance of an event and the retention and recollection of information. As noted in the Supplementary Material, the intensity of an event is a “more consistent predictor of autobiographical memory properties than was valence or the age of the memory” (Talarico et al., 2004, p. 2) and the effect of intensity on memory is independent of the valence of the emotion. Furthermore, Holland and Kensinger (2010) observed that emotional arousal and personal involvement in an event have a significant impact on “the likelihood that a vivid memory can be maintained over time” (Holland and Kensinger, 2010, p. 7). In other words, individuals retain and recall more information when an event is emotionally significant.

With respect to question 3, whether the network properties that predict the perceived threat of the coronavirus are consistent across samples, we are not able to make a definitive determination currently. Insofar the samples significantly differed in the PCT, it is not permissible to draw conclusions about whether the network measures are, or are not, equivalent across samples. Further data collection with additional independent samples is required.

A further consideration is the role of density in the PCT. As a measure of the CAM's interconnectedness, density was negatively and significantly correlated with the PCT in the combined sample but positively correlated with PCT in the German sample (while density was not significant in the Canadian sample its coefficient was also positive). Also challenging is that the interaction between density and valence of the central node was also positive and significant in the combined and German sample. This indicates that as graphs become denser and the central node becomes more positive, PCT increases. While this result is not easily interpreted, two statistical tendencies in the data are meaningful to its explanation. First, across the data we observed a large negative correlation between density and the number of nodes and links. This correlation indicates that dense CAMs have lower numbers of nodes (content) rather than a large number of highly interconnected nodes (Supplementary Tables 61–64). Second, the interaction between density and valence of the central node was negatively correlated with the percentage of positive nodes, and positively correlated with the number of negative nodes. In the German sample, these correlations reached significance at the 95% confidence level (Supplementary Tables 65–70). Counterintuitively, this suggests that CAMs with strongly positive central nodes contain a larger number of negative concepts while CAMs with strongly negative central nodes contain a larger number of positive concepts. While they did not reach significance, as we expected, the percentage of positive nodes in the German sample was negatively correlated with the PCT while the percentage of negative nodes was positively correlated with PCT (Supplementary Tables 71–76). This positive interaction between density and valence with PCT was contrary to expectations as the valence of the central node was positively correlated with the average valence of the CAM which, as shown, was negatively correlated with PCT (Supplementary Tables 77–79). This relationship held across all samples and reached significance at the 95% confidence level in the combined and Canadian sample. This result is difficult to interpret purely on the basis of an analysis of CAM network properties. To explore this interaction effect, the content of the central nodes would need to be considered. One may assume that the source of the interaction is that the central nodes may disproportionately feature negative concepts such as “coronaavirus” while the CAMs themselves are populated with positive concepts such as “working from home” or vice versa. While a content analysis of all CAM nodes is beyond the scope of this study, a categorization of the central nodes is available in the Supplementary Tables 82, 83. Among the seven inductively created categories (*Coronavirus*; *Quarantine*; *Restrictions*; *Isolation*; *Stress*; *Freetime*; *Other*), the category “Coronavirus" is the most frequent in both samples, i.e., a large proportion of participants placed the term “Pandemic” or “Coronavirus” directly in the middle. Other frequent central nodes were “Quarantine” (Canada) and “Restrictions” (Germany), assumably also negative concepts. Future studies should elaborate more the valence of central nodes, as well as the content of all concepts of the CAMs. We suggest that CAMs are a very helpful tool to dive deeper into a research topic such as the corona virus pandemic and allow participants to elaborate complex impressions, including affective connotations while at the same time CAMs give the possibility to collect large samples and apply network analyses. Yet, to enable this more work is needed to develop a content wise automated analyses which can be directly applied to CAM data.

## Conclusion

We explored Cognitive-Affective Maps (CAMs) as a tool to capture peoples' experiences with the ongoing coronavirus pandemic and to predict their perceived coronavirus threat (PCT) across the Canadian and German samples. Our findings showed consistent and significant relationships between emotional and latent network variables and PCT in both samples. The average valance of a CAM reliably predicted PCT. Also, there were consistent correlations between PCT and the latent structural variables centrality and density. The Canadian and German samples differed in their PCT value, which prevents a statement about the equality of correlation with network properties across samples. Further studies are needed to make clearer statements about differences and similarities of network measures between samples. It is currently difficult to draw specific psychological conclusions from the network data of the CAMs. In the future, methods should be found to automatically evaluate the contents of the CAMs and to relate them to the network properties. Yet, we suggest that CAMs can bridge several gaps between qualitative and quantitative methods. Unlike when using quantitative tools (e.g., questionnaires), participants' answers are not restricted by response items as participants are free to incorporate any thoughts and feelings on the given topic. Furthermore, as compared to traditional qualitative measures, such as structured interviews, CAMs may better enable researcher to objectively assess and integrate the substance of a shared experience for large samples of participants.

## Data Availability Statement

The raw data supporting the conclusions of this article is available through OSF: https://osf.io/8mxcz/?view_only=750d8048ed6a4c629d03f11bcc03c454.

## Ethics Statement

Ethical review and approval was not required for the study on human participants in accordance with the local legislation and institutional requirements. The patients/participants provided their written informed consent to participate in this study.

## Author Contributions

JM was primarily responsible for the statistical analysis and results. LR was primarily responsible for the study programming and implementation. CR was responsible for the administration of the Valence software tool. AK was responsible for overseeing the project development and direction. All authors contributed to the study design, statistical analysis, and preparation of the manuscript.

## Conflict of Interest

The authors declare that the research was conducted in the absence of any commercial or financial relationships that could be construed as a potential conflict of interest.

## Footnotes

^1^https://cam1.psychologie.uni-freiburg.de/users/loginpage?next=/.

^2^Conway et al. (2020) 3-item Perceived Coronavirus Threat (PCT) Questionnaire: (1) Thinking about the coronavirus (COVID-19) makes me feel threatened; (2) I am afraid of the coronavirus (COVID-19); (3) I am stressed around other people because I worry I'll catch the coronavirus (COVID-19).

^3^Blavatnik School of Government, University of Oxford. Data as of March 25. Data for the most recent seven days may not yet reflect government response changes implemented during that period. © FT Retrieved from: https://ig.ft.com/coronavirus-lockdowns/.
