# Supplementary material for: A Novel Network Approach to Capture Cognition and Affect: COVID-19 Experiences in Canada and Germany
Source: Front Psychol. 2021 Jun 11;12:663627. doi: 10.3389/fpsyg.2021.663627 (PMC8226173; doi:10.3389/fpsyg.2021.663627)

Electronic Supplementary Material

Table 1

General Linear Regression Model (GLM) with Robust Confidence Intervals in Combined Sample

Variable	Model 0	Model 1	Model 2	Model 3
Average Valence	-0.217**<br>(0.088)	-0.281**<br>(0.091)	-0.231**<br>(0.084)	-0.151***<br>(0.012)
Central Node Valence				
Centrality	0.166***<br>(0.10)	0.078***<br>(0.006)		0.093***<br>(0.012)
Density	-0.174**<br>(0.058)	-0.159**<br>(0.070)		-0.217***<br>(0.059)
Diameter			-0.104†<br>(0.058)	
Number Nodes			0.204***<br>(0.009)	
Number Links				
Number Dashed				
Number Solid				
Percentage Ambivalent				
Percentage Negative	-0.120<br>(0.078)	-0.165**<br>(0.078)	-0.114<br>(0.072)	
Percentage Neutral				
Percentage Positive				
Triadic Closure				0.109**<br>(0.055)
Age			-0.054**<br>(0.024)	-0.048**<br>(0.021)
Education			-0.017<br>(0.026)	-0.002**<br>(0.021)
Gender				
Female			-0.360**	-0.283

			(0.135)	(0.181)
Non-binary			-1.084***	-1.391**
			(0.272)	(0.508)
Pref Not Say			1.503***	1.478***
			(0.255)	(0.226)
Country		-0.654***	-0.652***	-0.634***
		(0.028)	(0.030)	(0.048)
Need for Affect				0.030
				(0.099)
Need for Structure				0.209***
				(0.025)
constant	0.000	0.339	0.800	0.649
	(0.303)	(0.014)	(0.146)	(0.351)
N	193	193	193	193
Residual *df*	192	192	192	192
Scale parameter	0.987	0.890	0.855	0.821
Residual D	0.967	0.867	0.810	0.770
AIC	2.809	2.700	2.633	2.581

Note. Correlation between network measures and a standardized measure of the perceived threat of coronavirus in combined Canadian and German samples.

†p < 0.100. **p < 0.050. ***p < 0.001.

Table 2

General Linear Regression Model (GLM) with Robust Confidence Intervals in Separated Samples

Variable	Model 1 Canada	Model 1 Germany	Model 2 Canada	Model 2 Germany	Model 3 Canada	Model 3 Germany
Average Valence	-0.418** (0.132)		-0.399** (0.139)			
Central Node Valence						
Centrality	-0.151 (0.122)					
Density$_c$		1.102† (0.448)		0.332† (0.193)		0.342† (0.192)
Diameter	-0.326** (0.127)		-0.223† (0.117)		0.188 (0.119)	
Number Nodes	0.649** (0.225)	1.102** (0.448)	-0.913*** (0.303)	0.444** (0.177)	-0.760*** (0.316)	0.429** (0.168)
Number Links	-0.490** (0.225)		1.092** (0.275)		0.931** (0.080)	
Number Dashed						
Number Solid						
Percentage Ambivalence						
Percentage Negative	-0.292** (0.123)		-0.295** (0.128)		-0.097 (0.080)	
Percentage Neutral						
Percentage Positive					-0.259** (0.091)	
Triadic Closure			0.211† (0.116)		0.185 (0.115)	
Age			-0.111† (0.067)	-0.099 (0.126)	-0.101 (0.063)	-0.064 (0.132)
Education			-0.046	0.001	-0.046	0.087

			(0.060)	(0.160)	(0.059)	(0.144)
Gender						
Female			-0.415**	-0.239	-0.340**	-0.099
			(0.171)	(0.219)	(0.168)	(0.220)
Non-binary			-1.843***	-0.763†	-2.083***	-0.879**
			(0.338)	(0.398)	(0.415)	(0.385)
Pref Not Say			1.821***		1.752***	
			(0.343)		(0.359)	
Need for Affect					0.163†	-0.070
					(0.095)	(0.094)
Need for Structure					0.160†	0.242**
					(0.091)	(0.093)
constant	0.371	-0.333	1.165	0.079	1.102	-0.593
	(0.090)	(0.098)	(0.453)	(1.063)	(0.448)	(0.971)
N	93	100	93	100	93	100
Residual *df*	86	97	83	94	81	92
Scale parameter	0.754	0.953	0.671	0.968	0.654	0.920
Residual D	0.754	0.953	0.655	0.957	0.638	0.910
AIC	2.628	2.819	2.516	2.853	2.508	2.820

Note. Correlation between network measures and a standardized measure of the perceived threat of coronavirus in combined Canadian and German samples.

†p < 0.100. **p < 0.050. ***p < 0.001.

Table 3

General Linear Statistical Model (GLM): Interaction between Emotional and Latent Network and the Standardized Measure of the Perceived Threat of Coronavirus. Combined Sample

Variable	Coefficient	*SD*	Z	P>z	95% CI
Average Valence	-0.228	0.161	-1.410	0.158	[-0.544, 0.089]
Centrality	0.674	0.080	8.380	0.0001***	[0.516, 0.831]
Central Node Value	0.043	0.017	2.560	0.010**	[0.010, 0.076]
Density Log	-0.418	0.151	-2.760	0.006**	[-0.715, -0.122]
Number Nodes Log	-0.354	0.012	-28.690	0.0001***	[-0.378, -0.329]
Number Links Log	0.321	0.189	1.700	0.090†	[-0.049, 0.691]
Percentage Negative	-0.131	0.080	-1.630	0.103	[-0.289, 0.026]
Percentage Ambivalent	-0.015	0.001	-14.280	0.0001***	[-0.017, -0.013]
Centrality # Central Node Value	0.234	0.024	9.550	0.0001***	[0.186, 0.282]
Density# Central Node Value	0.086	0.044	1.950	0.051†	[0.000, 0.172]
Country	-0.674	0.005	-132.820	0.000	[-0.684, -0.664]
constant	-0.016	0.051	-0.310	0.753	[-0.115, 0.083]
N					193
Residual *df*					192
Scale P					0.842
Residual D					0.794
AIC					2.613

Note. †p < 0.100. **p < 0.050. ***p < 0.001.

Table 4

General Linear Statistical Model: Interaction between Emotional and Latent Network and the Standardized Measure of the Perceived Threat of Coronavirus. Canadian Sample

Variable	Coefficient	*SD*	Z	P>z	95% CI
Average Valence	-0.371	0.118	-3.140	0.002**	[-0.603, -0.139]
Diameter	-0.198	0.098	-2.030	0.042**	[-0.390, -0.007]
Number Links	-0.865	0.282	-3.070	0.002**	[-1.417, -0.312]
Number Solid	0.364	0.178	2.050	0.040**	[0.016, 0.712]
Number Nodes	0.823	0.201	4.090	0.0001***	[0.428, 1.217]
Percentage Negative	-0.219	0.110	-1.980	0.048**	[-0.435, -0.002]
Central Node Value	0.102	0.100	1.020	0.307	[-0.094, 0.299]
Number Nodes#Percentage Negative	-0.269	0.092	-2.920	0.004**	[-0.451, -0.088]
Number Nodes# Central Node Value	-0.337	0.088	-3.820	0.0001***	[-0.510, -0.164]
constant	0.351	0.087	4.030	0.0001	[0.180, 0.521]
N					93
Residual *df*					83
Scale P					0.662
Residual D					0.662
AIC					2.526

Note. †p < 0.100. **p < 0.050. ***p < 0.001.

Table 5

General Linear Statistical Model: Interaction between Emotional and Latent Network and the Standardized Measure of the Perceived Threat of Coronavirus. German Sample

Variable	Coefficient	*SD*	Z	P>z	95% CI
Density	0.388	0.250	1.550	0.121	[-0.103, 0.878]
Number Nodes	0.566	0.221	2.560	0.010**	[0.133, 0.998]
Triadic Closure	-0.044	0.120	-0.360	0.717	[-0.279, 0.192]
Central Node Value	0.100	0.099	1.000	0.315	[-0.095, 0.294]
Density Log# Central Node Value	0.793	0.255	3.110	0.002**	[0.293, 1.293]
Number Nodes Log#Central Node Value	0.495	0.229	2.160	0.031**	[0.046, 0.944]
Triadic Closure#Central Node Value	-0.530	0.120	-4.430	0.0001***	[-0.765, -0.296]
constant	-0.341	0.089	-3.820	0.0001	[-0.516, -0.166]
N					100
Residual *df*					92
Scale P					0.881
Residual D					0.881
AIC					2.788

Note. †p < 0.100. **p < 0.050. ***p < 0.001.

Summary of Network Measures

Table 6

Full summary of network measures

Measure	Description	Scale
Emotional Network Properties		
Average Valence	Overall emotional value of a CAM based on the sum normalized valence of the individual nodes. Neutral and ambivalent nodes are scored as zero.	-3-3
Percentage Ambivalent	Percentage of nodes in a CAM that are ambivalent.	0-1
Percentage Negative	Percentage of nodes in a CAM that are negative.	0-1
Percentage Neutral	Percentage of nodes in a CAM that are neutral.	0-1
Percentage Positive	Percentage of nodes in a CAM that are positive.	0-1
Central Node Valence	Emotional value of each CAMs central node. 7-point negative to positive scale, neutral and ambivalent are scored as the midpoint.	1-7
Latent Network Properties		
Centrality	Measure of degree centrality, defined here as the number of links (solid and dashed links and arrows) on a node normalized by the total number of possible links. If a CAM contains several central nodes (equivalent central nodes) then it shows the grouping of important concepts.	
Density	The number of links a CAM has, divided by the total possible links a CAM could have.	0-1
Diameter	Maximum distance from one node to another - longest path in the graph.	0-8
Number of Nodes	Total number of nodes.	3-34
Number of Links	Total number of links.	3-40
Number of Supporting Links	Total number of supporting links (solid links).	0-40

Number of Contradicting Links	Total number of contradicting links (dashed links).	0-18
Triadic Closure	Total number of triangles (three nodes connected with each other by links) divided by total number of possible triangles.	0-1

Exploratory Question Rational

Exploratory Questions:

1. Do the emotional network properties of CAMs (e.g., average valence and valence of central node) predict the perceived threat of the coronavirus?
2. Do the latent network properties of CAMs (e.g., density, diameter, closure) predict the perceived threat of the coronavirus?
3. If so, to what extent are the network properties that predict the perceived threat of the coronavirus consistent across samples?

The rationale for the first exploratory question is based on the fact that the coronavirus pandemic is having negative consequences for billions of people. A structural feature of CAMs is that individuals can indicate the emotional valence of each concept they include in their network. Two expectations that follow from these negative consequences are that: 1) individuals whose lives have been disproportionally negatively affected by the coronavirus should be both more likely to associate it with negative concepts and experiences, and; 2) individuals who have had more negative experiences should be more likely to perceive the coronavirus as threatening. Consequently, if CAMs do meaningfully represent individuals' thoughts and experiences with an event (the coronavirus pandemic), then these (negative) associations should be encoded into their

CAMs and should be predictive of the PCT. In other words, the network measures capturing CAMs emotional-properties, average valence or percentage of negative nodes, should correlate with the PCT.

Regarding the second exploratory question, we are interested in the potential meaning of latent properties of individual CAMs. In the network's literature, there is little individual-level research on cognition which can inform us about whether or how the structured relationships between concepts may predict an individual's thoughts or perceptions. To our knowledge, this is the first study to empirically investigate this question. However, within the study of memory, research demonstrates an association between the significance of an event and the recollection and retention of information. For example, the intensity of an event is a "more consistent predictor of autobiographical memory properties than was valence or the age of the memory" (Talarico et al., 2004, p. 2) and the effect of intensity on memory is independent of the valence of the emotion. Furthermore, Holland and Kensigner (2010) observed that emotional arousal and personal involvement in an event have a significant impact on "the likelihood that a vivid memory can be maintained over time" (Holland & Kensigner, 2010, p. 7). In other words, individuals retain and recall more information when an event is emotionally significant. As we assume that the emotional impact of the coronavirus on people's lives is predominantly negative, we expect the information encoding during the CAM exercise to be predominantly negative, (and positively correlated with the PCT, see exploratory question 1). Following the argument that the emotional intensity of an event is a predictor of memory, a relevant question is whether the PCT will be also correlated with the density, the volume and interconnectedness of CAMs? As the valence of the network can also indicate emotional intensity, we explore this question by also conducting an additional analysis to explore whether there is a significant interaction effect between the network properties density and valence.

The interpretation of density and its relation to PCT is particularly interesting. Density, a measure of network connectivity, is an important property within the study of networks. However, applied to the study of human thought and experience the interpretation of density is not straightforward. Anecdotally people may conflate density with complexity or sophistication. However, the connectedness of a network does not necessarily demonstrate these characteristics, as networks with a fewer absolute number of nodes and links can still be considered high in density. Furthermore, in the context of drawing a CAM, a highly dense network often suggests that the outcomes of an event are not dependent on a small number of nodes (concepts) or relationships. Finally, within the computational study of networks, high density is often associated with a redundancy of information. While unnecessary for the operation of computers, in humans these redundancies may represent an effective mechanism to store and recall important information (Berntsen, 2001; Bohanek et al., 2005; Holland & Kensinger, 2010; Reisberg et al., 1988; Waters & Leeper, 1936). Consequently, in the context of the coronavirus pandemic studying the relationship between density and perception is significant to future research on human thought and experience.

Finally, predictive reliability in CAMs' network properties is important to the assessment of CAMs as a research tool. When assessing multiple covariates, caution is required when distinguishing between statistical and meaningful significant relationship as the odds of randomly finding a statistically significant effect (a false positive) increase with the number of tested variables. Important to the interpretation of results is that the same variables or families of variables are consistently retained across the modeling process (see analysis section for details on modeling process).

Correlation between Date of Data Collection and PCT

Table 7

General Linear Statistical Model. Correlation between perceived threat of coronavirus and the dates of data collection. Canadian sample

Variable	Coefficient	SD	Z	P>z	95% CI
Collection Date	-0.158	0.182	-0.87	0.386	[-0.515, 0.199]
Constant	0.357	0.118	3.01	0.003	[0.124, 0.589]
N					93
Residual df					91
Scale P					0.836
Residual D					0.836
AIC					2.680

Note. AIC = Akaike information criterion; CI = confidence interval. Table uses robust standard errors.

Table 8

General Linear Statistical Model. Correlation between perceived threat of coronavirus and the dates of data collection. German sample

Variable	Coefficient	SD	Z	P>z	95% CI
Collection Date	0.032	0.222	0.14	0.885	[-0.403, 0.468]
constant	-0.303	0.116	-2.62	0.009	[-0.529, -0.076]
N					100
Residual df					93
Scale P					0.9971
Residual D					0.997
AIC					2.855

Note. AIC = Akaike information criterion; CI = confidence interval. Table uses robust standard errors.

Demographics

Table 9

Reported Age: By Country

Age	Canada	Germany	Total
18-25	0 (0.00%)	1 (1.00%)	1 (0.52%)
26-32	42 (45.16%)	56 (56.00%	98 (50.78%)
33-39	27 (29.03%)	29 (29.00%)	56 (29.02%)
40-46	18 (19.35%)	12 (12.00%	30 (15.54%)
47-53	2 (2.15%)	1 (1.00%)	3 (1.55%)
54-60	2 (2.15%)	1 (1.00%)	3 (1.55%)
61-67	1 (1.08%)	0 (0.00%)	1 (0.52%)
68+	1 (1.08%)	0 (0.00%	1 (0.52%
Total	93	100	193

Table 10

Mean Reported Age Category: By Country

Age	N	M	SD	Min	Max
Canada	93	2.946	1.174	2	8
Germany	100	2.590	0.830	1	6

Table 11

Reported Education: By Country

Education	Canada	Germany	Total
Grade School	0 (0.00%)	0 (0.00%)	0 (0.00%)
Some High School	3 (3.23%)	0 (0.00%)	3 (1.55%)
High School	14 (15.04%)	0 (0.00%)	14 (7.25%)
Trade School	3 (3.23%)	10 (10.00%)	13 (6.74%)
Some College	19 (20.43%)	10 (10.00%)	29 (15.03%)
College Degree	36 (38.71%)	77 (77.00%)	113 (58.33%)
Graduate School	14 (15.05%)	1 (1.00%)	15 (7.77%)
Doctorate	4 (4.30%)	2 (2.00%)	6 (3.11%)
Total	93	100	193

Table 12

Mean Reported Education: By Country

Education	N	M	SD	Min	Max
Canada	93	5.387	1.475	2	8
Germany	100	5.750	0.730	4	8

Table 13

Reported Gender: By Country

Gender	Canada	Germany	Total
Female	41 (44.09%)	34 (34.00%)	75 (38.86%)
Male	50 53.76%	65 (65.00%)	115 (59.59%)
Non-binary	1 (1.08%)	1 (1.00%)	2 (1.04%)
Prefer Not to Say	1 (1.08%)	0 (0.00%)	1 (0.52%)
Total	93	100	193

Table 14

Reported Religious Importance: By Country

Gender	Canada	Germany	Total
Not important at all/Don't know	35 (37.63%)	54 (54.00%)	89 (46.11%)
Not very important	17 (18.28%)	22 (22.00%)	39 (20.11%)
Somewhat important	27 (29.03%)	20 (20.00%)	47 (24.35%)
Very important	14 (15.05%)	4 (4.00%)	18 (9.33%)
Total	93	100	193

Table 15

Mean Religiosity: By Country

Religiosity	N	M	SD	Min	Max
Canada	93	2.215	1.112	1	4
Germany	100	1.740	0.917	1	4

Table 16

Reported Ethnic Identity in the Canadian Sample

Ethnicity	Frequency	Percent	Cumulative
White	51	26.42%	26.42%
Non-white	142	73.58%	100.00%
Total	93	100	100

Table 17

Reported Ethnic Identity in the German Sample

German Birth Parents	Frequency	Percent	Cumulative
Both Parents	76	76.00%	76.00%
Mother	5	5.00%	81.00%
Father	7	7.00%	88.00%
Neither	12	12.00%	100.00%
Total	100	100	100

Measures

Table 18

Perceived Threat of Coronavirus (PCT): Scores on the standardized composite measure of perceived threat of coronavirus by country

ZPCTScale	Canada	Germany	Total
-1.671	2 (2.15%)	11 (11.00%)	13 (6.74%)
-1.458	1 (1.08%)	6 (6.00%)	7 (3.63%)
-1.244	5 (5.38%)	8 (8.00%)	13 (6.74%)
-1.031	2 (2.15%)	12 (12.00%)	14 (7.25%)
-.817	5 (5.38%)	5 (5.00%)	10 (5.18%)
-.604	5 (5.38%)	4 (4.00%)	9 (4.66%)
-.390	7 (7.53%)	4 (4.00%)	11 (5.70%)
-.177	5 (5.38%)	6 (6.00%)	11 (5.70%)
.038	4 (4.30%)	11 (11.00%)	15 (7.77%)
.250	5 (5.38%)	4 (4.00%)	9 (4.66%)
.463	10 (10.75%)	3 (3.00%)	13 (6.74%)
.677	9 (9.68%)	9 (9.00%)	18 (9.33%)
.890	11 (11.83%)	7 (7.00%)	18 (9.33%)
1.104	9 (9.68%)	4 (4.00%)	13 (6.74%)
1.317	4 (4.30%)	2 (2.00%)	6 (3.11%)
1.531	3 (3.23%)	1 (1.00%)	4 (2.07%)
1.744	3 (3.23%)	2 (2.00%)	5 (2.59%)
1.958	2 (2.15%)	0 (0.00%)	2 (1.04%)
2.171	1 (1.08%)	1 (1.00%)	2 (1.04%)
Total	93	100	193

Table 19

Mean scores on standardized composite measure of the Perceived Threat of Coronavirus (PCT) by country

ZPCTScale	N	M	SD	Min	Max
Canada	93	0.314	0.912	-1.671	2.171
Germany	100	-0.292	0.994	-1.671	2.171

Table 20

Alpha PCTQ1, PCTQ2, PCTQ3, Canadian sample

Average interitem covariance	1.571
Number of items in the scale	3
Scale reliability coefficient	0.7745

Table 21

Alpha PCTQ1, PCTQ2, PCTQ3, German sample

Average interitem covariance	2.127
Number of items in the scale	3
Scale reliability coefficient	0.8835

Table 22

Social Psychological Measurements of the Impact of COVID-19

Measure	Dimension	Number of Questions
Perceived Coronavirus Threat	Experience threat of corona virus	3
Restriction Scale	Support for government restrictions on citizens to stop viral spread	2
Punishment Scale	Support for government to punish citizens who violated social distance rules	2
Reactance Scale	Angry towards government resulting from restriction of freedoms	2
Research Scale	Support for government funded research on virus	2
Stimulus Scale	Support for government to provide stimulus money to support individuals	2
News Exposure Scale	Extent to which a participant is watching COVID related news	2
Psychological Impact Scale	Negative psychological effects of the virus	2
Financial Impact Scale	Negative financial effects of the virus	2
Informational Contamination Scale	Distrust in information from Federal Government	2
Proximity to Others Scale	Close proximity to other diagnosed with the virus	2
Personal Diagnoses Scale	Whether they have been diagnosed or display symptoms of the virus	3

Table 23

Alpha scores for all additional measures

Measure		Canada	Germany
Financial	Average interitem covariance	2.558	3.501
	Number of items in the scale	2	2
	Scale reliability coefficient	0.766*	0.847**
Information	Average interitem covariance	1.452	1.703
	Number of items in the scale	2	2
	Scale reliability coefficient	0.758*	0.725*
News	Average interitem covariance	1.727	1.748
	Number of items in the scale	2	2
	Scale reliability coefficient	0.740*	0.717*
Proximity	Average interitem covariance	0.354	0.771
	Number of items in the scale	2	2
	Scale reliability coefficient	0.427	0.542
Psychic	Average interitem covariance	1.804	1.787
	Number of items in the scale	2	2
	Scale reliability coefficient	0.743*	0.736*
Punish	Average interitem covariance	1.679	2.316
	Number of items in the scale	2	2
	Scale reliability coefficient	0.802**	0.891**
Reactance	Average interitem covariance	1.653	1.671
	Number of items in the scale	2	2
	Scale reliability coefficient	0.902***	0.800**
Research	Average interitem covariance	1.024	0.875
	Number of items in the scale	2	2
	Scale reliability coefficient	0.682	0.605
Resources	Average interitem covariance	1.823	2.646
	Number of items in the scale	2	2
	Scale reliability coefficient	0.791*	0.857**
Restrictions	Average interitem covariance	0.752	0.742
	Number of items in the scale	2	2
	Scale reliability coefficient	0.656	0.537
Stimulus	Average interitem covariance	1.205	0.471
	Number of items in the scale	2	2
	Scale reliability coefficient	0.755*	0.432
Symptoms	Average interitem covariance	0.581	0.829
	Number of items in the scale	3	3
	Scale reliability coefficient	0.581	0.536

Note: All variables are standardized with a mean of 0.

* good reliability. ** strong reliability. ***very strong reliability.

Table 24

Pearson's Correlations between Perceived Threat of Coronavirus and Other Response Scales

	Canada	Germany
Financial	0.1628<br>0.1189	0.1212<br>0.2296
Information	-0.0665<br>0.5266	-0.0981<br>0.3314
News	0.3927***<br>0.0001	0.2774**<br>0.0052
Proximity	0.0052<br>0.9608	0.0964<br>0.3401
Psychic	0.3483***<br>0.0006	0.2890**<br>0.0035
Punish	0.2082***<br>0.0452	0.2969**<br>0.0027
Reactance	-0.1305<br>0.2125	-0.1081<br>0.2845
Research	0.2431**<br>0.0188	0.3167**<br>0.0013
Resources	0.2093**<br>0.0441	0.1377<br>0.1719
Restrictions	0.3961***<br>0.0001	0.2182**<br>0.0292
Stimulus	0.2554**<br>0.0135	0.055<br>0.5869
Symptoms	-0.158<br>0.1305	0.0026<br>0.9795

Note. All variables are standardized with a mean of 0.

** $p < 0.050$. *** $p < 0.001$.

Table 25

General Linear Statistical Model. Summary of all measures which show a significant correlation with the perceived threat of coronavirus. Canadian sample

Variable	Coefficient	SD	Z	P>z	95% CI
News	0.289	0.076	3.810	0.0001***	[0.140, 0.437]
Psychic	0.348	0.078	4.460	0.0001***	[0.195, 0.500]
Restriction	0.402	0.059	6.760	0.0001***	[0.285, 0.518]
Constant	0.238	0.074	3.220	0.001	[0.093, 0.384]
N					93
Residual df					89
Scale P					0.501
Residual D					0.501
AIC					2.189

Note. AIC = Akaike information criterion; CI = confidence interval. Table uses robust standard errors. *** p < 0.001.

Table 26

General Linear Statistical Model. Summary of all measures which show a significant correlation with the perceived threat of coronavirus. German sample.

Variable	Coef.	Std.	Z	P>z	95% CI
News	0.192	0.092	2.100	0.036**	[0.013, 0.372]
Psych	0.274	0.108	2.550	0.011**	[0.063, 0.485]
Punish	0.213	0.076	2.810	0.005**	[0.065, 0.361]
Research	0.236	0.079	2.970	0.003**	[0.080, 0.392]
cons	-0.240	0.094	-2.550	0.011	[-0.425, -0.055]
N					100
Residual df					95
Scale P					0.749
Residual D					0.749
AIC					2.597

Note. AIC = Akaike information criterion; CI = confidence interval. Table uses robust standard errors. ** p < 0.050.

Table 27

Alpha scores for all additional scales

Scale		Canada	Germany
Need for Affect	Average interitem covariance	0.541	0.813
	Number of items in the scale	10	10
	Scale reliability coefficient	0.794*	0.830**
Need for Structure	Average interitem covariance	0.886	0.954
	Number of items in the scale	6	6
	Scale reliability coefficient	0.840**	0.806**
Cognitive Complexity	Average interitem covariance	1.438	1.504
	Number of items in the scale	2	2
	Scale reliability coefficient	0.786*	0.750*
Cognitive Simplicity	Average interitem covariance	0.995	1.016
	Number of items in the scale	2	2
	Scale reliability coefficient	0.507	0.569

* good reliability, ** strong reliability, *** very strong reliability.

Network Measures

Table 28

Summary of emotion-oriented network variable properties in by country

Variable	N	M	SD	Min	Max
Average Valence					
Canada	93.000	0.103	1.101	-2.392	2.592
Germany	100.000	-0.096	0.891	-2.676	2.037
PerAmb[a]					
Canada	93.000	-0.123	0.892	-0.950	2.603
Germany	100.000	0.115	1.083	-0.950	3.314
PerNeg[b]					
Canada	93.000	-0.039	1.082	-2.712	2.795
Germany	100.000	0.036	0.921	-1.961	2.695
PerNeut[c]					
Canada	93.000	-0.056	1.078	-1.000	6.252
Germany	100.000	0.052	0.924	-1.000	3.144
PerPos[d]					
Canada	93.000	0.166	1.069	-2.066	2.523
Germany	100.000	-0.154	0.910	-2.066	1.788
C-NodeVal[e]					
Canada	93.000	-0.059	1.052	-1.535	2.077
Germany	100.000	0.055	0.951	-1.535	2.077

[a]Percentage of ambivalent nodes. [b]Percentage of negative nodes. [c]Percentage of neutral nodes. [d]Percentage of positive nodes. [e]Central node value.

Table 29

Summary of latent network variable properties in by country

Variable	N	M	SD	Min	Max
Centrality					
Canada	93.000	0.249	1.073	-2.475	2.003
Germany	100.000	-0.232	0.870	-1.548	2.003
Density					
Canada	93.000	0.249	1.073	-2.475	2.003
Germany	100.000	-0.232	0.870	-1.548	2.003
Diameter					
Canada	93	-0.135	1.021	-2.430	2.926
Germany	100	0.125	0.968	-1.665	2.160
Number Nodes					
Canada	93.000	-0.199	0.973	-3.654	2.496
Germany	100.000	0.185	0.994	-2.360	2.262
Number Links					
Canada	93.000	-0.200	0.993	-3.290	1.965
Germany	100.000	0.186	0.975	-2.707	1.965
Number Dashed					
Canada	93.000	-0.294	0.921	-1.468	1.752
Germany	100.000	0.274	0.997	-1.468	2.033
Number Solid					
Canada	93.000	-0.059	1.010	-4.516	2.141
Germany	100.000	0.055	0.992	-3.274	2.005
Triadic Closure					
Canada	93	-0.811	1.046	-1.524	1.254
Germany	100	0.075	0.954	-1.524	1.138

Table 30

*Pearson's correlation between latent network measures. Combined sample. Significant correlations are marked by *.*

	Central Node	Density	Number Blocks	Number Links	Links Dashed	Links Solid	Triadic Closure	Diameter
Centrality	1							
Density	0.6812*<br>0.0001	1						
Number Blocks	-0.685*<br>0.0001	-0.845*<br>0.0001	1					
Number Links	-0.527*<br>0.0001	-0.509*<br>0.0001	0.878*<br>0.0001	1				
Links Dashed	-0.108<br>0.1334	-0.072<br>0.318	0.249*<br>0.0005	0.350*<br>0.0001	1			
Links Solid	-0.494*<br>0.0001	-0.490*<br>0.0001	0.770*<br>0.0001	0.834*<br>0.0001	-0.118<br>0.1029	1		
Triadic Closure	0.051<br>0.480	0.297*<br>0.0001	0.044<br>0.5398	0.372*<br>0.001	0.1515*<br>0.0367	0.295*<br>0.0001	1	
Diameter	-0.742<br>0.0001*	-0.600*<br>0.0001	0.709*<br>0.0001	0.604*<br>0.0001	0.139<br>0.0534	0.550*<br>0.0001	-0.029<br>0.6892	1

Note: As the measures are not all normally distributed caution is required when interpreting the statistical significance of the statistics.

Table 31

*Pearson's correlation between latent network measures. Canadian sample. Significant correlations are marked by **

	Central Node	Density	Number Blocks	Number Links	Links Dashed	Links Solid	Triadic Closure	Diameter
Centrality	1							
Density	0.665*<br>0.0001	1						
Number Blocks	-0.661*<br>0.0001	-0.823*<br>0.0001	1					
Number Links	-0.525*<br>0.0001	-0.505*<br>0.0001	0.872*<br>0.0001	1				
Links Dashed	-0.136<br>0.195	-0.117<br>0.264	0.180*<br>0.0005	0.188*<br>0.0001	1			
Links Solid	-0.437*<br>0.0001	-0.437*<br>0.0001	0.771*<br>0.0001	0.883*<br>0.0001	-0.205<br>0.049	1		
Triadic Closure	0.055<br>0.604	0.299*<br>0.0001	0.033<br>0.5398	0.356*<br>0.001	0.007<br>0.949	0.316*<br>0.002	1	
Diameter	-0.792<br>0.0001*	-0.595*<br>0.0001	0.729*<br>0.0001	0.609*<br>0.0001	0.052<br>0.618	0.550*<br>0.0001	-0.044<br>0.674	1

Note: As the measures are not all normally distributed caution is required when interpreting the statistical significance of the statistics.

Table 32

*Pearson's correlation between latent network measures. German sample. Significant correlations are marked by **

	Central Node	Density	Number Blocks	Number Links	Links Dashed	Links Solid	Triadic Closure	Diameter
Centrality	1							
Density	0.694*<br>0.0001	1						
Number Blocks	-0.689*<br>0.0001	-0.872*<br>0.0001	1					
Number Links	-0.485*<br>0.0001	-0.491*<br>0.0001	0.861*<br>0.0001	1				
Links Dashed	-0.054<br>0.591	0.034<br>0.741	0.229*<br>0.022	0.424*<br>0.0001	1			
Links Solid	-0.568*<br>0.0001	-0.536*<br>0.0001	0.779*<br>0.0001	0.817*<br>0.0001	-0.084<br>0.1029	1		
Triadic Closure	0.095<br>0.346	0.324*<br>0.0001	0.027<br>0.793	0.375*<br>0.001	0.267*<br>0.007	0.268*<br>0.007	1	
Diameter	-0.680*<br>0.0001	-0.593*<br>0.0001	0.680*<br>0.0001	0.582*<br>0.0001	0.158<br>0.117	0.546*<br>0.0001	-0.035<br>0.731	1

Note: As the measures are not all normally distributed caution is required when interpreting the statistical significance of the statistics.

Table 33

*Pearson's correlation between emotion-oriented network measures. Combined sample. Significant correlations are marked by **

	AvgVal[a]	PerPos[b]	PerNeg[c]	PerNeut[d]	PerAmb[e]	CNodeV[f]
AvgVal[a]	1					
PerPos[b]	0.8140*<br>0.0001	1				
PerNeg[c]	-0.8324*<br>0.0001	-0.5275*<br>0.0001	1			
PerNeut[d]	0.055<br>0.4477	-0.3679*<br>0.0001	-0.4143*<br>0.0001	1		
PerAmb[e]	0.0455<br>0.5299	-0.1826*<br>0.011	-0.2899*<br>0.0001	-0.1243<br>0.0851	1	
CNodeVal[f]	0.2571*<br>0.0003	0.1896*<br>0.0083	-0.2879*<br>0.0001	0.1056<br>0.144	0.0409<br>0.5721	1

Note: As the measures are not all normally distributed caution is required when interpreting the statistical significance of the statistics.
[a]Average valence. [b]Percentage of positive nodes. [c]Percentage of negative nodes. [d]Percentage of neutral nodes. [e]Percentage of ambivalent nodes. [f]Central node value.

Table 34

Pearson's correlation between emotion-oriented network measures. Canadian sample

	AvgVal[a]	PerPos[b]	PerNeg[c]	PerNeut[d]	PerAmb[e]	CNodeV[f]
AvgVal[a]	1					
PerPos[b]	0.8231*<br>0.0001	1				
PerNeg[c]	-0.8560*<br>0.0001	-0.5596*<br>0.0001	1			
PerNeut[d]	0.121<br>0.2478	-0.3219*<br>0.0017	-0.4808*<br>0.0001	1		
PerAmb[e]	-0.0095<br>0.9276	-0.2120*<br>0.0414	-0.185<br>0.0758	-0.1016<br>0.3323	1	
CNodeVal[f]	0.3627*<br>0.0004	0.2127*<br>0.0406	-0.4124*<br>0.0001	0.2238*<br>0.031	0.0669<br>0.524	1

Note: As the measures are not all normally distributed caution is required when interpreting the statistical significance of the statistics.
[a]Average valence. [b]Percentage of positive nodes. [c]Percentage of negative nodes. [d]Percentage of neutral nodes. [e]Percentage of ambivalent nodes. [f]Central node value.

Table 35

Pearson's correlation between emotion-oriented network measures. German sample

	AvgVal[a]	PerPos[b]	PerNeg[c]	PerNeut[d]	PerAmb[e]	CNodeV[f]
AvgVal[a]	1					
PerPos[b]	0.7988*<br>0.0001	1				
PerNeg[c]	-0.8035*<br>0.0001	-0.4889*<br>0.0001	1			
PerNeut[d]	-0.0201<br>0.8429	-0.4187*<br>0.0001	-0.3363*<br>0.0006	1		
PerAmb[e]	0.1244<br>0.2177	-0.1311<br>0.1934	-0.4061*<br>0.0001	-0.1627<br>0.1058	1	
CNodeVal[f]	0.1406<br>0.163	0.1885<br>0.0604	-0.144<br>0.153	-0.0424<br>0.6757	0.0076<br>0.9399	1

Note: As the measures are not all normally distributed caution is required when interpreting the statistical significance of the statistics.

[a]Average valence. [b]Percentage of positive nodes. [c]Percentage of negative nodes. [d]Percentage of neutral nodes. [e]Percentage of ambivalent nodes. [f]Central node value.

Table 36

Pearson's correlation between latent structure and emotion-oriented network measures. Combined sample

	Dens[a]	NumNo[b]	NumLi[c]	Triad[d]	Diam[e]	AvgVal[f]	PerPos[g]	PerNeg[h]	PerNeut[i]	PerAmb[j]
Dens[a]	1.00									
NumNo[b]	-.845*<br>0.0001	1.00								
NumLi[c]	-.509*<br>0.0001	0.878*<br>0.0001	1.00							
Triad[d]	0.297*<br>0.0001	0.044<br>0.540	0.372*<br>0.0001	1.00						
Diam[e]	-.600*<br>0.0001	0.709*<br>0.0001	0.604*<br>0.0001	-.029<br>0.689	1.00					
AvgVal[f]	0.028<br>0.704	-.074<br>0.305	-.065<br>0.369	-<br>0.062<br>0.391	-.062<br>0.390	1.00				
PerPos[g]	0.032<br>0.659	-.095<br>0.189	-.102<br>0.160	-.063<br>0.387	-.089<br>0.220	0.814*<br>0.0001	1.00			
PerNeg[h]	-.063<br>0.388	0.044<br>0.543	-.002<br>0.979	0.031<br>0.670	0.048<br>0.505	-.832*<br>0.0001	-.528*<br>0.0001	1.00		
PerNeut[i]	0.062<br>0.651	-.023<br>0.753	0.004<br>0.958	0.053<br>0.469	-.051<br>0.479	0.055<br>0.448	-.368*<br>0.0001	-.414*<br>0.0001	1.00	
PerAmb[j]	-.033<br>0.651	0.113<br>0.118	0.166*<br>0.021	0.082<br>0.260	0.137<br>0.057	0.046<br>0.530	-.183*<br>0.011	-.290*<br>0.0001	-.124<br>0.085	1.00
CNVal[k]	0.051<br>0.480	-0.053<br>0.461	-0.046<br>0.528	0.026<br>0.722	0.018<br>0.808	0.257*<br>0.0003	0.190*<br>0.008	-.288*<br>0.0001	0.106<br>0.144	0.041<br>0.572

Note: As the measures are not all normally distributed caution is required when interpreting the statistical significance of the statistics.

[a]Density. [b]Number of nodes. [c]Number of links. [d]Triadic closure. [e]Diameter. [f]Average valence. [g]Percentage of positive nodes. [h]Percentage of negative nodes. [i]Percentage of neutral nodes. [j]Percentage of ambivalent nodes. [k]Central node value.

Table 37

Pearson's correlation between latent structure and emotion-oriented network measures. Canadian sample

	Dens[a]	NumNo[b]	NumLi[c]	Triad[d]	Diam[e]	AvgVal[f]	PerPos[g]	PerNeg[h]	PerNeut[i]	PerAmb[j]
Dens[a]	1.00									
NumNo[b]	-.823*<br>0.0001	1.00								
NumLi[c]	-.505*<br>0.0001	0.887*<br>0.0001	1.00							
Triad[d]	0.299*<br>0.004	0.033<br>0.751	0.356*<br>0.0001	1.00						
Diam[e]	-.595*<br>0.0001	0.729*<br>0.0001	0.609*<br>0.0001	-.044<br>0.674	1.00					
AvgVal[f]	0.023<br>0.824	-.135<br>0.196	-.142<br>0.175	-<br>0.170<br>0.104	-.059<br>0.572	1.00				
PerPos[g]	-.006<br>0.957	-.124<br>0.236	-.158<br>0.131	-<br>.230*<br>0.026	-.094<br>0.372	0.823*<br>0.0001	1.00			
PerNeg[h]	-.105<br>0.316	0.151<br>0.148	0.112<br>0.286	0.045<br>0.671	0.035<br>0.740	-.856*<br>0.0001	-.560*<br>0.0001	1.00		
PerNeut[i]	0.145<br>0.167	-.106<br>0.311	-.056<br>0.593	0.096<br>0.359	-.006<br>0.952	0.121<br>0.248	-.322*<br>0.002	-.481*<br>0.0001	1.00	
PerAmb[j]	-.020<br>0.851	0.110<br>0.295	0.173<br>0.098	0.191<br>0.067	0.122<br>0.242	0.010<br>0.928	-.212*<br>0.041	-.185*<br>0.076	-.102<br>0.332	1.00
CNVal[k]	0.115<br>0.274	-.098<br>0.351	-.052<br>0.620	0.056<br>0.594	0.033<br>0.756	0.363*<br>0.0004	0.213*<br>0.041	-.412*<br>0.0001	0.224<br>0.031	0.067<br>0.524

Note: As the measures are not all normally distributed caution is required when interpreting the statistical significance of the statistics.

[a]Density. [b]Number of nodes. [c]Number of links. [d]Triadic closure. [e]Diameter. [f]Average valence. [g]Percentage of positive nodes. [h]Percentage of negative nodes. [i]Percentage of neutral nodes. [j]Percentage of ambivalent nodes. [k]Central node value.

Table 38

Pearson's correlation between latent structure and emotion-oriented network measures. German sample

	Dens[a]	NumNo[b]	NumLi[c]	Triad[d]	Diam[e]	AvgVal[f]	PerPos[g]	PerNeg[h]	PerNeut[i]	PerAmb[j]
Dens[a]	1.00									
NumNo[b]	-.862*<br>0.0001	1.00								
NumLi[c]	-.491*<br>0.0001	0.861*<br>0.0001	1.00							
Triad[d]	0.324*<br>0.001	0.027<br>0.793	0.375*<br>0.0001	1.00						
Diam[e]	-.593*<br>0.0001	0.680*<br>0.0001	0.582*<br>0.0001	-.035<br>0.731	1.00					
AvgVal[f]	0.005<br>0.964	0.031<br>0.757	0.064<br>0.529	0.090<br>0.374	-.039<br>0.702	1.00				
PerPos[g]	0.030<br>0.767	-.005<br>0.962	0.021<br>0.835	0.164<br>0.103	-.042<br>0.681	0.799*<br>0.0001	1.00			
PerNeg[h]	-.006<br>0.951	0.084<br>0.408	-.144<br>0.152	-.128<br>0.204	0.054<br>0.593	-.804*<br>0.0001	-.489*<br>0.0001	1.00		
PerNeut[i]	-.013<br>0.895	0.043<br>0.675	0.048<br>0.638	-.008<br>0.934	-.119<br>0.237	-.020<br>0.843	-.419*<br>0.0001	-.336*<br>0.0006	1.00	
PerAmb[j]	-.016<br>0.877	0.080<br>0.431	0.129<br>0.203	-.025<br>0.808	0.126<br>0.211	0.124<br>0.218	-.131<br>0.193	-.406*<br>0.0001	-.163<br>0.106	1.00
CNVal[k]	0.002<br>0.991	-.034<br>0.740	-.064<br>0.527	-.017<br>0.864	-.014<br>0.890	0.141<br>0.163	0.189<br>0.060	-.144<br>0.153	-.042<br>0.676	0.008<br>0.940

Note: As the measures are not all normally distributed caution is required when interpreting the statistical significance of the statistics.

[a]Density. [b]Number of nodes. [c]Number of links. [d]Triadic closure. [e]Diameter. [f]Average valence. [g]Percentage of positive nodes. [h]Percentage of negative nodes. [i]Percentage of neutral nodes. [j]Percentage of ambivalent nodes. [k]Central node value.

Summary Tables: Stepwise Regression Model

Table 39

General Linear Regression Model (GLM) with Robust Confidence Intervals. Combined sample

Variable	Model 1:	Model 2:	Model 3:	Model 4:
AvgVal[a]	-0.217**	-0.281**	-0.231**	-0.151***
	(0.088)	*(0.091)*	*(0.084)*	*(0.012)*
CNodeVal[b]				
Centrality	*0.166****	0.078***		0.093***
	(0.10)	(0.006)		(0.012)
Density$_c$	*-0.174***	-0.159**		-0.217***
	(0.058)	(0.070)		*(0.059)*
Diameter			-0.104†	
			(0.058)	
Number Nodes			0.204***	
			(0.009)	
Number Links				
Number Dashed				
Number Solid				
PerAmb[c]				
PerNeg[d]	-0.120	-0.165**	-0.114	
	(0.078)	(0.078)	(0.072)	
PerNeut[e]				
PerPos[f]				
Triadic Closure				0.109**
				(0.055)
Age			*-0.054***	*-0.048***
			(0.024)	*(0.021)*
Education			-0.017	-0.002**
			(0.026)	(0.021)
Gender				
Female			-0.360**	-0.283
			(0.135)	(0.181)
Non-binary			-1.084***	-1.391**
			(0.272)	(0.508)
Pref NotSay			1.503***	1.478***
			(0.255)	(0.226)

Country		-0.654***	-0.652***	-0.634***
		(0.028)	(0.030)	(0.048)
NFA[g]				0.030
				(0.099)
NFS[h]				0.209***
				(0.025)
constant	0.000	0.339	0.800	0.649
	(0.303)	*(0.014)*	*(0.146)*	*(0.351)*
N	193	193	193	193
Residual df	192	192	192	192
Scale parameter	0.987	0.890	0.855	0.821
Residual D	0.967	0.867	0.810	0.770
AIC	2.809	2.700	2.633	2.581

Note. AIC = Akaike information criterion; Residual D=Deviance adjust for degrees of freedom. In this model the Deviance and Pearson's scores are identical. Correlation between network measures and a standardized measure of the perceived threat of coronavirus in combined Canadian and German samples. Model network variables are selected on the basis of a stepwise selection process. Table uses cluster robust standard errors.

[a]Average valence. [b]Central node value. [c]Percentage of ambivalent nodes. [d]Percentage of negative nodes. [e]Percentage of neutral nodes. [f]Percentage of positive nodes. [g]Need for affect. [h]Need for Structure.

†p < 0.100. **p < 0.050. ***p < 0.001.

Table 40

General Linear Regression Model (GLM) with Robust Confidence Intervals. Separate samples

Variable	Model 1: Can	Model 1: Ger	Model 2: Can	Model 2: Ger	Model 3: Can	Model 3: Ger
AvgVal[a]	-0.418**		-0.399**			
	(0.132)		*(0.139)*			
CNodeVal[b]						
Centrality	*-0.151*					
	(0.122)					
Density$_{c}$		*0.342†*		0.332†		*0.342†*
		(0.194)		*(0.193)*		*(0.192)*
Diameter	-0.326**		-0.223†		*-0.188*	
	(0.127)		(0.117)		*(0.119)*	
Number Nodes	0.649**	0.450**	1.092**	0.444**	0.931**	0.429**
	(0.225)	(0.166)	(0.275)	(0.177)	(0.080)	(0.168)
Number Links	-0.490**		-0.913***		-0.760***	
	(0.225)		(0.303)		(0.316)	
Number Dashed						
Number Solid						
PerAmb[c]						
PerNeg[d]	-0.292**		-0.295**		-0.097	
	(0.123)		(0.128)		(0.080)	
PerNeut[e]						
PerPos[f]					-0.259**	
					(0.091)	
Triadic Closure			0.211†		0.185	
			(0.116)		(0.115)	
Age			-0.111†	-0.099	-0.101	-0.064
			(0.067)	(0.126)	(0.063)	(0.132)
Education			-0.046	0.001	-0.046	0.087
			(0.060)	(0.160)	(0.059)	(0.144)
Gender						
Female			*-0.415***	-0.239	-0.340**	-0.099
			(0.171)	(0.219)	(0.168)	(0.220)
Non-binary			-1.843***	*-0.763†*	-2.083***	-0.879**
			(0.338)	*(0.398)*	(0.415)	(0.385)
PrefNotSay			1.821***		1.752***	
			(0.343)		(0.359)	

NFA[g]					0.163†	-0.070
					(0.095)	(0.094)
NFS[h]					*0.160*†	0.242**
					(0.091)	(0.093)
constant	0.371	-0.333	1.165	0.079	1.102	-0.593
	(0.090)	(0.098)	(0.453)	(1.063)	(0.448)	(0.971)
N	93	100	93	100	93	100
Residual df	86	97	83	94	81	92
Scale parameter	0.754	0.953	0.671	0.968	0.654	0.920
Residual D	0.754	0.953	0.655	0.957	0.638	0.910
AIC	2.628	2.819	2.516	2.853	2.508	2.820

Note. AIC = Akaike information criterion; Residual D=Deviance adjust for degrees of freedom. In this model the Deviance and Pearson's scores are identical. Correlation between network measures and a standardized measure of the perceived threat of coronavirus in combined Canadian and German samples. Model network variables are selected on the basis of a stepwise selection process. Table uses cluster robust standard errors.

[a]Average valence. [b]Central node value. [c]Percentage of ambivalent nodes. [d]Percentage of negative nodes. [e]Percentage of neutral nodes. [f]Percentage of positive nodes. [g]Need for affect. [h]Need for Structure.

†p < 0.100. **p < 0.050. ***p < 0.001.

Full Statistics of Final Stepwise Models.
Model 1: Zero-order Correlations

Table 41

General Linear Statistical Model: Correlation between CAM network properties and the standardized measure of the perceived threat of coronavirus. Combined Canadian and German samples

Variable	Coefficient	SD	Z	P>z	95% CI
Average Valence	-0.217	0.088	-2.460	0.014**	[-0.390, -0.044]
Density	-0.174	0.058	-3.020	0.003**	[-0.288, -0.061]
Centrality	0.166	0.010	16.880	0.0001***	[0.146, 0.185]
Percentage Negative	-0.120	0.078	-1.540	0.124	[-0.273, 0.033]
constant	0.000	0.303	0.000	1.000	[-0.593, 0.593]
N					193
Residual df					192
Scale P					0.987
Residual D					0.967
AIC					2.809

Note. AIC = Akaike information criterion; CI = confidence interval; Residual D = Deviance adjust for degrees of freedom. In this model the Deviance and Pearson’s scores are identical. Network variables are selected on the basis of a stepwise exploratory variable selection process. Table uses cluster robust standard errors.
$^{**}p < 0.050$. $^{***}p < 0.001$.

Table 42

General Linear Statistical Model: Correlation between CAM network properties and the standardized measure of the perceived threat of coronavirus. Combined Canadian and German samples

Variable	Coefficient	SD	Z	P>z	95% CI
Average Valence	-0.281	0.091	-3.110	0.002**	[-0.459, -0.104]
Centrality	0.078	0.006	13.980	0.0001***	[0.067, 0.089]
Density	-0.159	0.070	-2.280	0.023**	[-0.295, -0.022]
Percentage Negative	-0.165	0.078	-2.120	0.034**	[-0.317, -0.012]
Country (Ger)	-0.654	0.028	-23.680	0.0001***	[-0.708, -0.600]
constant	0.339	0.014	23.680	0.000	[0.311, 0.367]
N					193
Residual df					192
Scale P					0.890
Residual D					0.867
AIC					2.700

Note. AIC = Akaike information criterion; CI = confidence interval; Residual D = Deviance adjust for degrees of freedom. In this model the Deviance and Pearson's scores are identical. Network variables are selected on the basis of a stepwise exploratory variable selection process. Table uses cluster robust standard errors.
**p < 0.050. ***p < 0.001.

Table 43

General Linear Statistical Model: Correlation between CAM network properties and the standardized measure of the perceived threat of coronavirus in the Canadian sample

Variable	Coefficient	SD	Z	P>z	95% CI
Average Valence	-0.418	0.132	-3.160	0.002**	[-0.678, -0.158]
Centrality	-0.151	0.122	-1.240	0.215	[-0.390, 0.088]
Diameter	-0.326	0.127	-2.560	0.010**	[-0.575, -0.077]
Number Nodes	0.649	0.251	2.590	0.010**	[0.158, 1.140]
Number Links	-0.490	0.225	-2.180	0.030**	[-0.931, -0.049]
Percentage Negative	-0.292	0.123	-2.370	0.018**	[-0.534, -0.050]
constant	0.371	0.090	4.100	0.000	[0.194, 0.548]
N					93
Residual df					86
Scale P					0.754
Residual D					0.754
AIC					2.628

Note. AIC = Akaike information criterion; CI = confidence interval; Residual D = Deviance adjust for degrees of freedom. In this model the Deviance and Pearson's scores are identical. Network variables are selected on the basis of a stepwise exploratory variable selection process. Table uses cluster robust standard errors.
$^{**}p < 0.050$.

Table 44

General Linear Statistical Model: Correlation between CAM network properties and the standardized measure of the perceived threat of coronavirus in the German sample.

Variable	Coefficient	SD	Z	P>z	95% CI
Density	1.102	0.448	1.760	0.078†	[-0.038, 0.723]
Number Nodes	1.102	0.448	2.720	0.007**	[0.126, 0.775]
cons	-0.333	0.098	-3.400	0.001	[-0.524, -0.141]
N					100
Residual df					97
Scale P					0.953
Residual D					0.953
AIC					2.819

Note. AIC = Akaike information criterion; CI = confidence interval; Residual D = Deviance adjust for degrees of freedom. In this model the Deviance and Pearson's scores are identical. Network variables are selected on the basis of a stepwise exploratory variable selection process. Table uses cluster robust standard errors.
†$p < 0.100$. $^{**}p < 0.050$.

Table 45

General Linear Statistical Model. Correlation between CAM network properties and the standardized measure of the perceived threat of coronavirus. Combined Canadian and German samples

Variable	Coefficient	SD	Z	P>z	95% CI
Average Valence	-0.231	0.084	-2.760	0.006**	[-0.396, -0.067]
Diameter	-0.104	0.058	-1.790	0.073†	[-0.218, 0.010]
Number Nodes	0.204	0.009	21.680	0.0001***	[0.185, 0.222]
Percentage Negative	-0.114	0.072	-1.590	0.111	[-0.254, 0.026]
Age	-0.054	0.024	-2.280	0.023**	[-0.101, -0.008]
Education	-0.017	0.026	-0.660	0.511	[-0.067, 0.033]
Gender					
Female	-0.360	0.135	-2.650	0.008**	[-0.625, -0.094]
Non-binary	-1.084	0.272	-3.990	0.0001***	[-1.616, -0.551]
PrefNotSay	1.503	0.255	5.890	0.0001***	[1.003, 2.003]
Country	-0.652	0.030	-21.500	0.0001***	[-0.712, -0.593]
constant	0.800	0.146	5.470	0.0001	[0.513, 1.086]
N					193
Residual df					192
Scale P					0.855
Residual D					0.810
AIC					2.633

Note. AIC = Akaike information criterion; CI = confidence interval; Residual D = Deviance adjust for degrees of freedom. In this model the Deviance and Pearson's scores are identical. Network variables are selected on the basis of a stepwise exploratory variable selection process. Table uses cluster robust standard errors.

†p < 0.100. **p < 0.050. ***p < 0.001.

Table 46

General Linear Statistical Model: Correlation between CAM network properties and the standardized measure of the perceived threat of coronavirus. Canadian sample

Variable	Coefficient	SD	Z	P>z	95% CI
Average Valence	-0.399	0.139	-2.870	0.004**	[-0.671, -0.127]
Diameter	-0.223	0.117	-1.900	0.057†	[-0.453, 0.007]
Number Nodes	-0.913	0.303	-3.010	0.0001***	[-1.507, -0.319]
Number Links	1.092	0.275	3.970	0.003**	[0.553, 1.632]
Percentage Negative	-0.295	0.128	-2.310	0.021**	[-0.546, -0.044]
Triadic Closure	0.211	0.116	1.830	0.068†	[-0.016, 0.438]
Age	-0.111	0.067	-1.650	0.100†	[-0.243, 0.021]
Education	-0.046	0.060	-0.780	0.437	[-0.163, 0.070]
Gender					
Female	-0.415	0.171	-2.430	0.015**	[-0.750, -0.080]
Non-binary	-1.843	0.338	-5.450	0.0001***	[-2.506, -1.180]
PrefNotSay	1.821	0.343	5.310	0.0001***	[1.150, 2.493]
constant	1.165	0.453	2.570	0.010	[0.277, 2.053]
N					93
Residual df					83
Scale P					0.671
Residual D					0.655
AIC					2.516

Note. AIC = Akaike information criterion; CI = confidence interval; Residual D = Deviance adjust for degrees of freedom. In this model the Deviance and Pearson's scores are identical. Network variables are selected on the basis of a stepwise exploratory variable selection process. Table uses cluster robust standard errors.

†p < 0.100. **p < 0.050. ***p < 0.001.

Table 47

General Linear Statistical Model: Correlation between CAM network properties and the standardized measure of the perceived threat of coronavirus. German sample

Variable	Coefficient	SD	Z	P>z	95% CI
Density	0.332	0.193	1.720	0.085†	[-0.046, 0.710]
Number Nodes	0.444	0.177	2.520	0.012**	[0.098, 0.790]
Age	-0.099	0.126	-0.790	0.431	[-0.345, 0.147]
Education	0.001	0.160	0.010	0.993	[-0.313, 0.315]
Gender					
Female	-0.239	0.219	-1.090	0.274	[-0.668, 0.189]
Non-binary	-0.763	0.398	-1.920	0.055†	[-1.543, 0.017]
PrefNotSay	-	-	-	-	-
constant	0.079	1.063	0.070	0.941	[-2.005, 2.162]
N					100
Residual df					94
Scale P					0.968
Residual D					0.957
AIC					2.853

Note. AIC = Akaike information criterion; CI = confidence interval; Residual D = Deviance adjust for degrees of freedom. In this model the Deviance and Pearson's scores are identical. Network variables are selected on the basis of a stepwise exploratory variable selection process. Table uses cluster robust standard errors.

†p < 0.100. **p < 0.050. ***p < 0.001.

Table 48

General Linear Statistical Model: Correlation between CAM network properties and the standardized measure of the perceived threat of coronavirus. Combined Canadian and German samples

Variable	Coefficient	SD	Z	P>z	95% CI
Average Valence	-0.151	0.012	-12.970	0.0001***	[-0.173, -0.128]
Centrality	0.093	0.012	7.650	0.0001***	[0.069, 0.117]
Density	-0.217	0.059	-3.700	0.0001***	[-0.333, -0.102]
Triadic Closure	0.109	0.055	1.990	0.047**	[0.002, 0.217]
Age	-0.048	0.021	-2.340	0.020**	[-0.088, -0.008]
Education	-0.002	0.057	-0.040	0.967	[-0.114, 0.109]
Gender					
Female	-0.283	0.181	-1.570	0.117	[-0.637, 0.071]
Non-binary	-1.391	0.508	-2.740	0.006**	[-2.387, -0.395]
PrefNotSay	1.478	0.226	6.530	0.0001**	[1.035, 1.922]
Country	-0.634	0.048	-13.320	0.0001***	[-0.727, -0.540]
Need for Affect	0.030	0.099	0.300	0.761	[-0.163, 0.223]
Need for Structure	0.209	0.025	8.500	0.0001***	[0.161, 0.257]
constant	0.649	0.351	1.850	0.064	[-0.039, 1.337]
N					193
Residual df					192
Scale P					0.821
Residual D					0.770
AIC					2.581

Note. AIC = Akaike information criterion; CI = confidence interval; Residual D = Deviance adjust for degrees of freedom. In this model the Deviance and Pearson’s scores are identical. Network variables are selected on the basis of a stepwise exploratory variable selection process. Table uses cluster robust standard errors.
**p < 0.050. ***p < 0.001.

Table 49

General Linear Statistical Model: Correlation between CAM network properties and the standardized measure of the perceived threat of coronavirus. Canadian sample

Variable	Coefficient	SD	Z	P>z	95% CI
Diameter	-0.188	0.119	-1.580	0.113	[-0.421, 0.045]
Number Nodes	-0.760	0.316	3.360	0.001***	[-1.380, -0.140]
Number Links	0.931	0.277	-2.400	0.016**	[0.388, 1.474
Percentage Negative	-0.097	0.080	-1.210	0.228	[-0.255, 0.061]
Percentage Positive	-0.259	0.091	-2.840	0.004**	[-0.437, -0.080]
Triadic Closure	0.185	0.115	1.610	0.108	[-0.041, 0.410]
Age	-0.101	0.063	-1.610	0.108	[-0.224, 0.022]
Education	-0.046	0.059	-0.770	0.440	[-0.162, 0.070]
Gender					
Female	-0.340	0.168	-2.030	0.043**	[-0.668, -0.011]
Non-binary	-2.083	0.415	-5.020	0.0001***	[-2.896, -1.269]
PrefNotSay	1.752	0.359	4.880	0.0001***	[1.048, 2.455]
Need for Affect	0.163	0.095	1.700	0.088†	[-0.024, 0.349]
Need for Structure	0.160	0.091	1.760	0.078†	[-0.018, 0.337]
constant	1.102	0.448	2.460	0.014	[0.224, 1.980]
N					93
Residual df					81
Scale P					0.654
Residual D					0.638
AIC					2.508

Note. AIC = Akaike information criterion; CI = confidence interval; Residual D = Deviance adjust for degrees of freedom. In this model the Deviance and Pearson's scores are identical. Network variables are selected on the basis of a stepwise exploratory variable selection process. Table uses cluster robust standard errors.
†p < 0.100. **p < 0.050. ***p < 0.001.

Table 50

General Linear Statistical Model: Correlation between CAM network properties and the standardized measure of the perceived threat of coronavirus. German sample

Variable	Coefficient.	SD	Z	P>z	95% CI
Density	0.342	0.192	1.780	0.075†	[-0.035, 0.719]
Number Nodes	0.429	0.168	2.550	0.011**	[0.100, 0.759]
Age	-0.064	0.132	-0.490	0.627	[-0.322, 0.194]
Education	0.087	0.144	0.600	0.546	[-0.195, 0.370]
Gender					
Female	-0.099	0.220	-0.450	0.654	[-0.530, 0.332]
Non-binary	-0.879	0.385	-2.280	0.023**	[-1.634, -0.124]
PrefNotSay	-	-	-	-	-
Need for Affect	-0.070	0.094	-0.740	0.457	[-0.253, 0.114]
Need for Structure	0.242	0.093	2.610	0.009**	[0.060, 0.423]
cons	-0.593	0.971	-0.610	0.542	[-2.496, 1.310]
N					100
Residual df					92
Scale P					0.920
Residual D					0.910
AIC					2.820

Note. AIC = Akaike information criterion; CI = confidence interval; Residual D = Deviance adjust for degrees of freedom. In this model the Deviance and Pearson's scores are identical. Network variables are selected on the basis of a stepwise exploratory variable selection process. Table uses cluster robust standard errors.

†p < 0.100. **p < 0.050.

Alternative Models

Table 51

General Linear Statistical Model: Correlation between CAM network properties and the standardized measure of the perceived threat of coronavirus. Alternative Model to the stepwise outcome, the network variable Average Valence is substituted for the variables percent negative and percent positive. Canadian sample

Variable	Coefficient	SD	Z	P>z	95% CI
Average Valence	-0.143	0.072	-1.980	0.047*	[-0.284, -0.002]
Diameter	-0.160	0.126	-1.270	0.206	[-0.407, 0.087]
Number Nodes	0.863	0.260	3.320	0.001***	[0.353, 1.372]
Number Links	-0.719	0.296	-2.430	0.015**	[-1.299, -0.140]
Triadic Closure	0.208	0.117	1.780	0.075†	[-0.021, 0.437]
Age	-0.075	0.071	-1.050	0.293	[-0.214, 0.065]
Education	-0.052	0.061	-0.850	0.396	[-0.172, 0.068]
Gender					
Female	-0.438	0.165	-2.650	0.008**	[-0.762, -0.114]
Non-binary	-1.932	0.367	-5.260	0.0001***	[-2.651, -1.212]
PrefNotSay	1.454	0.326	4.460	0.0001***	[0.816, 2.093]
Need for Affect	0.123	0.099	1.250	0.212	[-0.070, 0.317]
Need for Structure	0.156	0.092	1.700	0.089†	[-0.024, 0.336]
constant	1.090	0.454	2.400	0.016	[0.199, 1.981]
N					93
Residual df					82
Scale P					0.673
Residual D					0.657
AIC					2.528

Note. AIC = Akaike information criterion; CI = confidence interval; Residual D = Deviance adjust for degrees of freedom. In this model the Deviance and Pearson’s scores are identical. Network variables are selected on the basis of a stepwise exploratory variable selection process. Table uses cluster robust standard errors.
†p < 0.100. **p < 0.050. ***p < 0.001.

Table 52

General Linear Statistical Model: Correlation between CAM network properties and the standardized measure of the perceived threat of coronavirus. Alternative Model to the stepwise outcome, the network variable Average Valence is substituted for the variables percent positive. Canadian sample

Variable	Coefficient	SD	Z	P>z	95% CI
Average Valence	-0.397	0.138	-2.860	0.004**	[-0.668, -0.125]
Diameter	-0.196	0.120	-1.640	0.100	[-0.431, 0.038]
Number Nodes	0.966	0.274	3.530	0.000***	[0.429, 1.503]
Number Links	-0.787	0.310	-2.540	0.011**	[-1.395, -0.180]
Percentage Negative	-0.296	0.131	-2.270	0.023**	[-0.553, -0.040]
Triadic Closure	0.193	0.116	1.660	0.097	[-0.035, 0.420]
Age	-0.117	0.067	-1.750	0.080†	[-0.247, 0.014]
Education	-0.044	0.060	-0.740	0.462	[-0.162, 0.074]
Gender					
Female	-0.373	0.166	-2.250	0.024**	[-0.698, -0.048]
Non-binary	-1.988	0.376	-5.280	0.000***	[-2.725, -1.250]
PrefNotSay	1.710	0.348	4.920	0.000***	[1.028, 2.392]
Need for Affect	0.133	0.097	1.360	0.173	[-0.058, 0.324]
Need for Structure	0.151	0.093	1.630	0.104	[-0.031, 0.333]
constant	1.149	0.455	2.520	0.012	[0.257, 2.041]
N					93
Residual df					81
Scale P					0.656
Residual D					0.640
AIC					2.510

Note. AIC = Akaike information criterion; CI = confidence interval; Residual D = Deviance adjust for degrees of freedom. In this model the Deviance and Pearson's scores are identical. Network variables are selected on the basis of a stepwise exploratory variable selection process. Table uses cluster robust standard errors.
†p < 0.100. **p < 0.050. ***p < 0.001.

Perceived Coronavirus Threat

Table 53

General Linear Statistical Model. Difference in the perceived coronavirus threat between Canada and Germany

Variable	Coefficient	SD	Z	P>z	95% CI
Germany	-0.606	0.137	-4.43	0.0001***	[-0.875, -0.338]
constant	0.314	0.094	3.33	0.001	[0.129, 0.499]
N					193
Residual df					191
Scale P					0.912
Residual D					0.912
AIC					2.757

Note. AIC = Akaike information criterion; CI = confidence interval. Table uses cluster robust standard errors.
***p < 0.001.

Discussion Regressions

Table 54

General Linear Statistical Model. Interaction between average valence of the CAM and country on the perceived threat of coronavirus.

Variable	Coefficient	SD	Z	P>z	95% CI
AvgVal[a]	-0.169	0.0001	-1.34^{15}	0.0001***	[-0.169, -0.169]
Country	-0.634	0.0001	-3.4^{14}	0.0001***	[-0.634, -0.634]
Country##AvgVal	0.063	0.0001	3.3^{14}	0.0001***	[0.063, 0.063]
cons	0.332	0.0001	2.3^{14}	0.0001	[0.332, 0.332]
N					193
Residual df					193
Scale P					0.901
Residual D					0.882
AIC					2.712

Note. AIC = Akaike information criterion; CI = confidence interval. Table uses cluster robust standard errors.
[a]AvgVal = Average valence.
***p < 0.001.

Table 55

General Linear Statistical Model. Interaction between percentage of positive nodes and country on the perceived threat of coronavirus.

Variable	Coefficient	SD	Z	P>z	95% CI
PerPos[a]	-0.198	0.0001	-2.2^{14}	0.0001***	[-0.198, -0.198]
Country	-0.034	0.0001	-2.9^{14}	0.0001***	[-0.646, -0.646]
Country##PerPos	0.151	0.0001	1.7^{14}	0.0001***	[0.151, 0.151]
constant	0.347	0.0001	2.0^{14}	0.0001	[0.347, 0.347]
N					193
Residual df					193
Scale P					0.899
Residual D					0.881
AIC					2.711

Note. AIC = Akaike information criterion; CI = confidence interval. Table uses cluster robust standard errors.
[a]PerPos = Percentage of positive nodes.
***p < 0.001.

Table 56

General Linear Statistical Model. Interaction between percentage of ambivalent nodes and country on the perceived threat of coronavirus.

Variable	Coefficient	SD	Z	P>z	95% CI
PerAm[a]	0.045	0.0001	2.8^{14}	0.0001***	[0.045, 0.045]
Country	-0.616	0.0001	-3.3^{14}	0.0001***	[-0.616, -0.616]
Country##PerAm	-0.010	0.0001	1.62^{13}	0.0001***	[-0.010, -0.010]
constant	0.320	0.0001	2.2^{14}	0.0001	[0.320, 0.320]
N					193
Residual df					193
Scale P					0.921
Residual D					0.902
AIC					2.734

Note. AIC = Akaike information criterion; CI = confidence interval. Table uses cluster robust standard errors.
[a]PerAm = Percentage of ambivalent nodes.
***p < 0.001.

Table 57

General Linear Statistical Model. Interaction between central node valence and country on the perceived threat of coronavirus.

Variable	Coefficient	SD	Z	P>z	95%
[a]C-NodeVal	0.017	0.0001	4.5^{14}	0.0001***	[0.017, 0.017]
Country	-0.603	0.0001	-3.3^{14}	0.0001***	[-0.603, -0.603]
Country## C-Node Val	-0.104	0.0001	6.7^{14}	0.0001***	[-0.104, -0.104]
constant	0.315	0.0001	2.3^{14}	0.0001	[0.315, 0.315]
N					193
Residual df					193
Scale P					0.918
Residual D					0.899
AIC					2.732

Note. AIC = Akaike information criterion; CI = confidence interval. Table uses cluster robust standard errors.
[a]C-NodeVal = Central node value.
***p < 0.001.

Table 58

General Linear Statistical Model. Interaction between percentage of negative nodes and country on the perceived threat of coronavirus.

Variable	Coefficient	SD	Z	P>z	95% CI
PerNeg[a]	0.098	0.0001	4.0^{15}	0.0001***	[0.098, 0.098]
Country	-0.612	0.0001	-3.0^{14}	0.0001***	[-0.612, -0.612]
Country##PerNeg	-0.052	0.0001	-1.0^{15}	0.0001***	[-0.052, -0.052]
constant	0.318	0.0001	2.1^{14}	0.0001	[0.318, 0.318]
N					193
Residual df					193
Scale P					0.916
Residual D					0.897
AIC					2.729

Note. AIC = Akaike information criterion; CI = confidence interval. Table uses cluster robust standard errors.
[a]PerNeg = Percentage of negative nodes.
***p < 0.001.

Table 59

General Linear Statistical Model. Interaction between percentage of neutral nodes and country on the perceived threat of coronavirus.

Variable	Coefficient	SD	Z	P>z	95% CI
PerNeutr	0.080	0.0001	5.3^{14}	0.0001***	[0.080, 0.080]
Country	-0.609	0.0001	-3.2^{14}	0.0001***	[-0.609, --0.609]
Country##PerNeutr	-0.116	0.0001	-4.5^{14}	0.0001***	[-0.116, -0.116]
constant	0.319	0.0001	1.42^{15}	0.0001	[0.319, 0.319]
N					193
Residual df					193
Scale P					0.918
Residual D					0.898
AIC					2.731

Note. AIC = Akaike information criterion; CI = confidence interval. Table uses cluster robust standard errors.
[a]PerNeutr = Percentage of neutral nodes.
***p < 0.001.

Table 60

Skewness and kurtosis tests for normality in the emotional network measures

Variable	N	Pr Skewness	SD	Adj Chi(2)	Prob>Chi(2)
Average Valence					
Canada	93	0.726	0.169	2.07	0.356
Germany	100	0.202	0.338	2.61	0.271
Central Node Value					
Canada	93	0.841	0.042	4.29	0.117
Germany	100	0.453	0.595	0.86	0.650
Percentage Ambivalent					
Canada	93	0.004	0.782	7.41	0.025
Germany	100	0.001	0.675	9.76	0.008
Percentage Negative					
Canada	93	0.770	0.731	0.20	0.903
Germany	100	0.189	0.808	1.83	0.401
Percentage Neutral					
Canada	93	0.001	0.001	62.80	0.001
Germany	100	0.001	0.086	16.38	0.003
Percentage Positive					
Canada	93	0.349	0.087	3.92	0.141
Germany	100	0.330	0.034	5.31	0.070

Table 61

General Linear Statistical Model. Correlation between number of nodes and the density of a network. Canadian Sample

Variable	Coefficient	SD	Z	P>z	95% CI
Number Nodes	-0.850	0.056	-15.26	0.0001***	[-0.959, -0.741]
constant	-0.034	0.061	-0.55	0.584	[-0.154, 0.087]
N					93
Residual df					91
Scale P					0.330
Residual D					0.330
AIC					1.750

Note. AIC = Akaike information criterion; CI = confidence interval. Table uses cluster robust standard errors.
***p < 0.001.

Table 62

General Linear Statistical Model. Correlation between number of nodes and the density of a network. German Sample

Variable	Coefficient	SD	Z	P>z	95% CI
Number Nodes	-0.853	0.054	-15.94	0.0001***	[-0.958, -0.748]
constant	0.032	0.054	0.59	0.554	[-0.074, 0.138]
N					100
Residual df					98
Scale P					0.252
Residual D					0.252
AIC					1.478

Note. AIC = Akaike information criterion; CI = confidence interval. Table uses cluster robust standard errors.
***p < 0.001.

Table 63

General Linear Statistical Model. Correlation between number of links and the density of a network. Canadian Sample

Variable	Coefficient	SD	Z	P>z	95% CI
Number Links	-0.511	0.102	-5.02	0.0001***	[-0.711, -0.311]
constant	0.033	0.095	0.35	0.725	[-0.153, 0.219]
N					93
Residual df					91
Scale P					0.761
Residual D					0.761
AIC					2.586

Note. AIC = Akaike information criterion; CI = confidence interval. Table uses cluster robust standard errors.
***p < 0.001.

Table 64

General Linear Statistical Model. Correlation between number of links and the density of a network. German Sample

Variable	Coefficient	SD	Z	P>z	95% CI
Number Links	-0.495	0.088	-5.60	0.0001***	[-0.668, -0.323]
constant	-0.034	0.086	-0.39	0.693	[-0.203, 0.135]
N					100
Residual df					98
Scale P					0.742
Residual D					0.742
AIC					2.559

Note. AIC = Akaike information criterion; CI = confidence interval. Table uses cluster robust standard errors.
***p < 0.001.

Table 65

General Linear Statistical Model. Interaction between density (Dens) and the valence of the central node (C-NodeVl) and the percentage of positive nodes. Combined sample

Variable	Coefficient	SD	Z	P>z	95% CI
Dens	0.053	0.059	0.90	0.370	[-0.062, 0.167]
C-NodeV	0.192	0.030	6.43	0.0001***	[0.133, 0.250]
Dens##C-NodeV	-0.097	0.096	-1.01	0.314	[-0.285, 0.091]
constant	0.005	0.164	0.03	0.976	[-0.317, 0.327]
N					193
Residual df					192
Scale P					0.970
Residual D					0.955
AIC					2.797

Note. AIC = Akaike information criterion; CI = confidence interval. Table uses cluster robust standard errors.
***p < 0.001.

Table 66

General Linear Statistical Model. Interaction between density (Dens) and the valence of the central node (C-NodeV) and the percentage of positive nodes. Canadian sample

Variable	Coefficient	Std.	Z	P>z	95% CI
Dens	-0.027	0.106	-0.25	0.801	[-0.233, 0.180]
C-NodeV	0.223	0.114	1.96	0.050**	[-0.0001, 0.446]
Dens##C-NodeV	-0.020	0.080	-0.25	0.803	[-0.178, 0.137]
cons	0.185	0.112	1.64	0.100	[-0.036, 0.405]
N					93
Residual df					89
Scale P					1.126
Residual D					1.126
AIC					2.999

Note. AIC = Akaike information criterion; CI = confidence interval. Table uses cluster robust standard errors.
**p < 0.050.

Table 67

General Linear Statistical Model. Interaction between density (Dens) and the valence of the central node (D-NodeV) and the percentage of positive nodes. German sample

Variable	Coefficient	SD	Z	P>z	95% CI
Dens	0.096	0.104	0.93	0.353	[-0.107, 0.299]
C-NodeV	0.152	0.087	1.74	0.082†	[-0.019, 0.324]
Dens##C-NodeV	-0.218	0.100	-2.18	0.029**	[-0.414, -0.022]
constant	-0.152	0.926	-1.64	0.102	[-0.333, 0.030]
N					100
Residual df					96
Scale P					0.785
Residual D					0.785
AIC					2.635

Note. AIC = Akaike information criterion; CI = confidence interval. Table uses cluster robust standard errors.
†p < 0.100. **p < 0.050.

Table 68

General Linear Statistical Model. Interaction between density (Dens) and the valence of the central node (C-NodeVal) and the percentage of negative nodes. Combined sample

Variable	Coefficient	SD	Z	P>z	95% CI
Dens	-0.095	0.011	-8.440	0.0001***	[-0.117, -0.073]
C-NodeVal	-0.290	0.156	-1.860	0.063†	[-0.596, 0.015]
Dens##C-NodeV	0.150	0.124	1.210	0.227	[-0.093, 0.393]
constant	-0.008	0.044	-0.170	0.863	[-0.094, 0.079]
N					193
Residual df					192
Scale P					0.908
Residual D					0.894
AIC					2.731

Note. AIC = Akaike information criterion; CI = confidence interval. Table uses cluster robust standard errors.
†p < 0.100. ***p < 0.001.

Table 69

General Linear Statistical Model. Interaction between density (Dens) and the valence of the central node (C-NodeV) and the percentage of negative nodes Canadian sample

Variable	Coefficient	SD	Z	P>z	95% CI
Dens	-0.080	0.094	-0.860	0.392	[-0.265, 0.104]
C-NodeV	-0.428	0.101	-4.240	0.0001***	[-0.626, -0.230]
Den##C-NodeV	0.061	0.067	0.900	0.368	[-0.071, 0.193]
constant	-0.060	0.105	-0.570	0.569	[-0.267, 0.147]
N					93
Residual df					89
Scale P					0.997
Residual D					0.997
AIC					2.876

Note. AIC = Akaike information criterion; CI = confidence interval. Table uses cluster robust standard errors.
***p < 0.001.

Table 70

General Linear Statistical Model. Interaction between density (Dens) and the valence of the central node (C-NodeV) and the percentage of negative nodes German sample

Variable	Coefficient	SD	Z	P>z	95% CI
Dens	-0.109	0.108	-1.010	0.312	[-0.321, 0.102]
C-NodeV	-0.097	0.084	-1.150	0.249	[-0.263, 0.068]
Den##C-NodeV	0.329	0.094	3.490	0.0001***	[0.144, 0.514]
constant	0.030	0.091	0.330	0.744	[-0.148, 0.208]
N					100
Residual df					96
Scale P					0.772
Residual D					0.772
AIC					2.618

Note. AIC = Akaike information criterion; CI = confidence interval. Table uses cluster robust standard errors.
***p < 0.001.

Table 71

General Linear Statistical Model. Correlation between the percentage of positive nodes (PerPos) and the perceived threat of coronavirus. Combined Sample

Variable	Coefficient	SD	Z	P>z	95% CI
PerPos	-0.132	0.074	-1.770	0.077†	[-0.277, 0.014]
Country	-0.649	0.024	-27.280	0.0001***	[-0.695, -0.602]
constant	0.336	0.012	27.280	0.0001	[0.312, 0.360]
N					193
Residual df					193
Scale P					0.900
Residual D					0.891
AIC					2.727

Note. AIC = Akaike information criterion; CI = confidence interval. Table uses cluster robust standard errors.
†p < 0.100. ***p < 0.001.

Table 72

General Linear Statistical Model. Correlation between the percentage of positive nodes (PerPos) and the perceived threat of coronavirus. Canadian Sample

Variable	Coefficient	SD	Z	P>z	95% CI
PerPos	-0.198	0.084	-2.360	0.018**	[-0.362, -0.034]
constant	0.347	0.092	3.770	0.0001	[0.167, 0.527]
N					93
Residual df					91
Scale P					0.796
Residual D					0.796
AIC					2.631

Note. AIC = Akaike information criterion; CI = confidence interval. Table uses cluster robust standard errors.
†p < 0.100. **p < 0.050.

Table 73

General Linear Statistical Model. Correlation between the percentage of positive nodes (Perpos) and the perceived threat of coronavirus. German Sample

Variable	Coefficient	SD	Z	P>z	95% CI
PerPos	-0.047	0.112	-0.420	0.677	[-0.266, 0.173]
constant	-0.299	0.101	-2.950	0.003	[-0.498, -0.101]
N					100
Residual df					98
Scale P					0.996
Residual D					0.996
AIC					2.853

Note. AIC = Akaike information criterion; CI = confidence interval. Table uses cluster robust standard errors.

Table 74

General Linear Statistical Model. Correlation between the percentage of negative nodes (PerNeg) and the perceived threat of coronavirus. Combined Sample

Variable	Coefficient	SD	Z	P>z	95% CI
PerNeg	0.075	0.026	2.910	0.004**	[0.024, 0.125]
Country	-0.612	0.002	-316.950	0.0001**	[-0.616, -0.608]
constant	0.317	0.001	316.950	0.0001	[0.315, 0.319]
N					193
Residual df					192
Scale P					0.912
Residual D					0.902
AIC					2.740

Note. AIC = Akaike information criterion; CI = confidence interval. Table uses cluster robust standard errors.
**$p < 0.050$.

Table 75

General Linear Statistical Model. Correlation between the percentage of negative nodes (PerNeg) and the perceived threat of coronavirus. Canadian Sample

Variable	Coef.	Std.	Z	P>z	95% CI
PerNeg	0.098	0.074	1.310	0.190	[-0.048, 0.243]
constant	0.318	0.094	3.390	0.001	[0.134, 0.502]
N					93
Residual df					91
Scale P					0.830
Residual D					0.830
AIC					2.673

Note. AIC = Akaike information criterion; CI = confidence interval. Table uses cluster robust standard errors.

Table 76

General Linear Statistical Model. Correlation between the percentage of negative nodes (PerNeg) and the perceived threat of coronavirus. German Sample

Variable	Coefficient	SD	Z	P>z	95% CI
PerNeg	0.045	0.127	0.360	0.720	[-0.203, 0.293]
constant	-0.294	0.099	-2.970	0.003	[-0.488, -0.100]
N					100
Residual df					98
Scale P					0.996
Residual D					0.996
AIC					2.853

Note. AIC = Akaike information criterion; CI = confidence interval. Table uses cluster robust standard errors.

Table 77

General Linear Statistical Model. Correlation between the valence of the central node (C-NodeV) and average valence of the CAM. Combined Sample

Variable	Coefficient	SD	Z	P>z	95% CI
C-NodeV	0.264	0.123	2.130	0.033**	[0.022, 0.506]
Country	-0.228	0.014	-16.290	0.0001**	[-0.256, -0.201]
constant	0.118	0.007	16.290	0.0001	[0.104, 0.132]
N					193
Residual df					192
Scale P					0.931
Residual D					0.921
AIC					2.761

Note. AIC = Akaike information criterion; CI = confidence interval. Table uses cluster robust standard errors
**p < 0.050.

Table 78

General Linear Statistical Model. Correlation between the valence of the central node (C-NodeV) and average valence of the CAM. Canadian Sample

Variable	Coefficient	SD	Z	P>z	95% CI
C-NodeV	0.380	0.100	3.790	0.0001***	[0.183, 0.576]
constant	0.125	0.107	1.170	0.241	[-0.084, 0.334]
N					93
Residual df					91
Scale P					1.065
Residual D					1.065
AIC					2.922

Note. AIC = Akaike information criterion; CI = confidence interval. Table uses cluster robust standard errors
***p < 0.001.

Table 79

General Linear Statistical Model. Correlation between the valence of the central node (C-NodeV) and average valence of the CAM. German Sample

Variable	Coefficient	SD	Z	P>z	95% CI
C-NodeV	0.132	0.101	1.300	0.193	[-0.066, 0.330]
constant	-0.103	0.087	-1.180	0.240	[-0.274, 0.069]
N					100
Residual df					98
Scale P					0.785
Residual D					0.785
AIC					2.616

Note. AIC = Akaike information criterion; CI = confidence interval. Table uses cluster robust standard errors

Power Analysis Tables

Table 80

One-sided post-hoc power analysis based on Model 2 in the independent Canadian and German Samples.

Variable	Coefficient	SD	P>z	Required Sample	Achieved Power
Canada				N=93	
Average Valence	0.399	0.139	**	*n*=35	λ=*0.99*
Diameter	0.223	0.117	†	*n*=120	λ=*0.70*
Number of Nodes	0.913	0.223	***	*n*=5	λ=*0.99*
Number of Links	1.092	0.275	**	*n*=8	λ=*0.99*
Percentage Negative	0.295	0.128	**	*n*=67	λ=*0.90*
Triadic Closure	0.211	0.116	†	*n*=135	λ=*0.67*
Germany				N=100	
Density	0.332	0.193	†	*n*=52	λ=*0.96*
Number of Nodes	0.444	0.177	**	*n*=27	λ=*0.99*

Note. α =0.05, λ=0.80. †p < 0.100. **p < 0.050. ***p < 0.001. *Power analysis is reported for results that reach marginal significance or better.* Power analysis was implemented through Stata.

Table 81

One-sided post-hoc power analysis based on the Interaction Model for the independent Canadian and German Samples.

Variable	Coefficient	SD	P>z	Required Sample	Achieved Power
Canada				N=93	
Number of Nodes	0.823	0.201	***	*n*=6	*λ=0.99*
Percentage Negative	0.219	0.110	**	*n*=125	*λ=0.68*
Number of Nodes # Percentage Negative	0.269	0.092	**	*n*=81	*λ=0.84*
Number of Nodes # Central Node Value	0.337	0.088	***	*n=50*	*λ=0.96*
Germany				N=100	
Number of Nodes	0.566	0.221	**	*n=15*	*λ=0.99*
Density # Central Node Value	0.793	0.255	**	*n*=6	*λ=0.99*
Number of Nodes # Central Node Value	0.495	0.229	**	*n*=21	*λ=0.99*
Triadic Closure # Central Node Value	0.530	0.120	***	*n*=18	*λ=0.99*

Note. α =0.05, λ=0.80. †p < 0.100. **p < 0.050. ***p < 0.001. *Power analysis is reported for results that reach marginal significance or better.* Power analysis was implemented through Stata.

Central Nodes

Table 82

Categorization of the CAMs' Central Nodes in Both Samples

Category	Examples	Frequency	
		Canada	Germany
Coronavirus	Corona; COVID; Pandemic	42	40
Quarantine	Quarantine; Staying/Working from Home	17	3
Restrictions	Restrictions; Shut/Lockdown; Less Leisure Activities	2	13
Isolation	Isolation; Distancing; not Seeing friends	4	4
Stress	Stress; Mental Health; Emotional State; Fear	8	8
Freetime	More Freetime; More Time for Myself/Family	2	4
Other	e.g. Job; Political Tension…	18	28

Table 83

Categorization of CAM's reported by Valence in Both Samples

Category	*Negative Canadian*	*Neutral Canadian*	*Positive Canadian*	*Negative German*	*Neutral German*	*Positive German*
Coronavirus	*19*<br>*61.29%*	*23*<br>*48.94%*	*0*<br>*0.00%*	*11*<br>*32.35%*	*29*<br>*55.77%*	*0*<br>*0.00%*
Quarantine	*2*<br>*6.45%*	*9*<br>*19.15%*	*6*<br>*40.00%*	*1*<br>*2.94%*	*2*<br>*3.85%*	*0*<br>*0.00%*
Restrictions	*2*<br>*6.45%*	*0*<br>*0.00%*	*0*<br>*0.00%*	*4*<br>*11.76%*	*7*<br>*13.46%*	*2*<br>*14.29%*
Isolation	*1*<br>*3.32%*	*2*<br>*4.26%*	*1*<br>*6.67%*	*3*<br>*8.82%*	*0*<br>*0.00%*	*1*<br>*7.14%*
Stress	*3*<br>*9.68%*	*3*<br>*6.38%*	*2*<br>*13.33%*	*4*<br>*11.76%*	*2*<br>*3.85%*	*2*<br>*14.29%*
Freetime	*0*<br>*0.00%*	*2*<br>*4.26%*	*0*<br>*0.00%*	*0*<br>*0.00%*	*0*<br>*0.00%*	*4*<br>*28.57%*
Other	*4*<br>*12.90%*	*8*<br>*17.02*	*6*<br>*40.00%*	*11*<br>*32.35%*	*12*<br>*23.08%*	*5*<br>*35.71%*
Total	*31*	*47*	*15*	*34*	*52*	*14*

Electronic Supplementary Material

Instructions

Double-click the picture to open the full instructions.

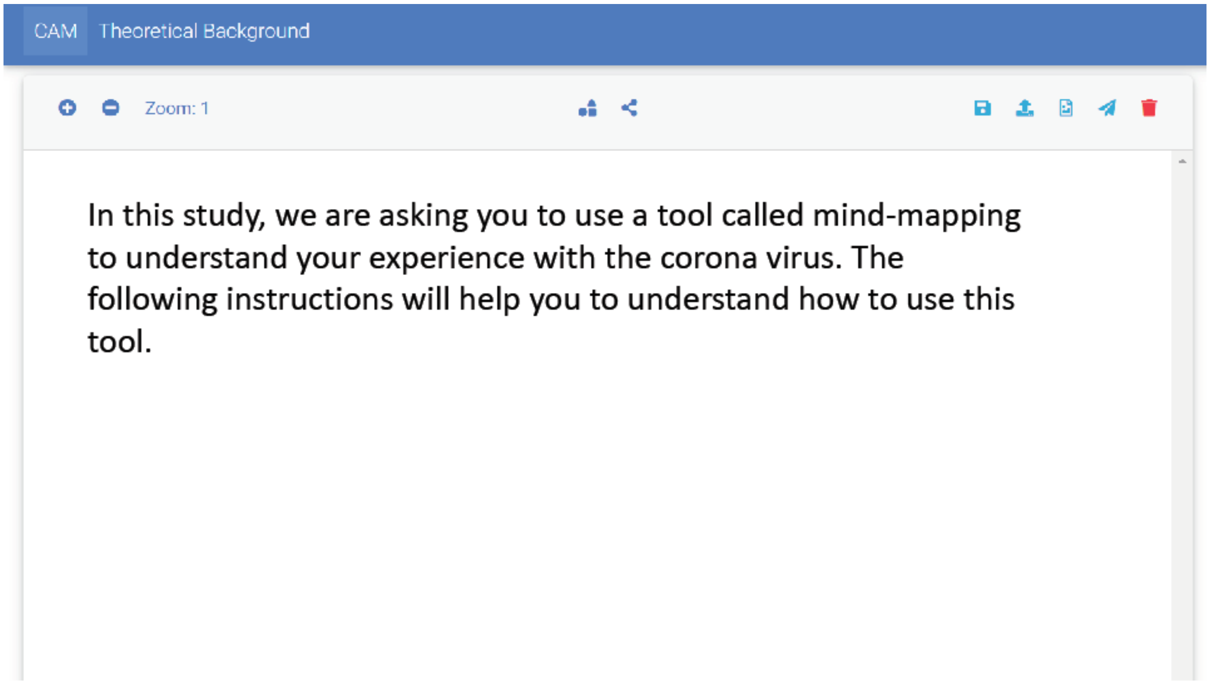

Supplement: Supplementary file 1 [file Data_Sheet_1.PDF]
